# Emerging Device Applications From Strong Light–Matter Interactions in 2D Materials

**DOI:** 10.1002/advs.202520976

**Published:** 2026-02-11

**Authors:** Janani Archana K, Kumar Shwetabh, Reyas Ali, Ramji Velayutham, Koustav Das, Abhishek Mondal, Prashant Kumar, Surendra B. Anantharaman

**Affiliations:** ^1^ Low‐dimensional Semiconductors Lab Department of Metallurgical and Materials Engineering Indian Institute of Technology Madras Chennai India; ^2^ School of Materials Science and Engineering Nanyang Technological University Singapore Singapore; ^3^ Center for 2D Materials Research and Innovation Indian Institute of Technology Madras Chennai India; ^4^ School of Interdisciplinary Studies (SIDiS) Indian Institute of Technology Madras Chennai India

**Keywords:** 2D materials, exciton, magnons, plasmons, polariton, optoelectronic devices

## Abstract

Two‐dimensional (2D) semiconductors provide a powerful platform for highly compact optoelectronic devices, spanning solar cells, photodetectors, light‐emitting diodes (LEDs), and lasers. In these materials, tightly bound excitons dominate key metrics such as absorption strength, quantum efficiency, response speed, and spectral purity. When driven into the strong light–matter coupling regime, excitons hybridize with cavity photons, plasmons, or magnons to form exciton‐, plasmon‐, and magnon‐polaritons, enabling engineered dispersion, low‐threshold lasing, ultrafast modulation, and enhanced nonlinear functionality within footprint‐limited architectures. Earlier reviews have focused on the fundamentals of strong coupling, band engineering, and realizing strong coupling with a variety of 2D materials. In this review, we will discuss different device architectures based on exciton and polaritons‐based systems, integrating 2D materials and heterostructures with dielectric cavities, metasurfaces, waveguides, and hybrid metal/2D magnet platforms. We emphasize design strategies based on the best figures of merit for solar cells, photodetectors, and lasers from exciton‐ and polariton‐based systems. Finally, we will discuss the routes to on‐chip integration of LEDs from all‐2D materials‐based devices for next‐generation photonic integrated circuits. Further, we will discuss advanced electron microscopy and nano‐imaging to map polaritonic fields and exciton distributions, linking nanoscale coupling to macroscopic device behavior and outlining a roadmap for next‐generation exciton/polariton devices.

## Graphical abstract

Two‐dimensional semiconductors enable extremely compact optoelectronic devices such as solar cells, sensors, LEDs, and lasers. Their strong light–matter interactions allow efficient light emission, detection, and energy conversion. This review article discusses the recent progress in integrating these materials with optical cavities and nanostructures to achieve low‐power, fast, and scalable photonic devices, paving the way for next‐generation on‐chip optical technologies.

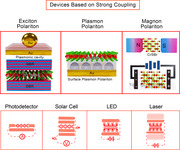

## 1 Introduction

Modern digital society relies heavily on electronic devices in daily life, including LEDs [1, 2, 3], lasers [4], photodetectors [5], and solar cells [6]. Silicon, the backbone of semiconductor technology, underpins applications ranging from solar cells for energy harvesting to transistors for display technologies. In contrast, compound semiconductors such as GaAs, InP, and GaN have revolutionized light emission. For example, InP laser diodes, widely used in fiber‐optic telecommunications, enable transmission at the 1550 nm (C‐band) wavelength with minimal loss [7]. A major advantage of these materials is the availability of mature fabrication processes, engineered charge injection and extraction layers, and reliable packaging [8].

While three‐dimensional semiconductors continue to dominate the market, low‐dimensional materials such as quantum dots [9, 10], nanowires [11, 12], and two‐dimensional semiconductors [13, 14, 15, 16, 17, 18] have emerged as promising alternatives, offering synergies between optical and electronic functionalities at smaller scales. Since the successful mechanical exfoliation of monolayer graphene and the synthesis of phase‐pure perovskite quantum dots, low‐dimensional semiconductors have been recognized for their disruptive potential [13, 14, 15, 16, 19, 20].

2D transition metal dichalcogenides (TMDs), represented as MX_2_, consist of transition metals (M = Mo, W) combined with chalcogens (X = S, Se, Te). These materials exhibit strong in‐plane covalent bonding and weak interlayer van der Waals interactions. Their bandgap is layer‐dependent, with multilayers typically supporting indirect excitons that transform into direct excitons in monolayers. By contrast, 2D metal halide perovskites exhibit bandgap dependence on the number of lead–iodide octahedral layers rather than overall thickness. In both TMDs and 2D perovskites, optical responses are governed by tightly bound electron–hole pairs, known as excitons. Upon illumination with photons of energy equal to or greater than the bandgap, excitons form with high binding energies (∼0.5 eV), opening new opportunities for next‐generation photonic and optoelectronic devices, including LEDs, solar cells, and photodetectors.

Previous reviews have largely focused on exciton‐based optoelectronic devices [21, 22, 23, 24, 25], while those on strong light–matter coupling (exciton–polaritons) have emphasized fundamental aspects, often with limited attention to device applications. For instance, reviews on Bose–Einstein condensation and exciton–polaritons in organic microcavities [26] and 2D perovskites [27, 28, 29] have mainly concentrated on low‐threshold lasers, offering little discussion of their practical implementation [30]. Devices beyond polariton lasers, such as polariton photodetectors, plexciton photodetectors, and polariton photovoltaics, remain underexplored. Moreover, critical topics such as electrically driven operation and dynamic manipulation of polaritons, which are essential for advanced optoelectronic technologies, are largely absent. Beyond exciton–polaritons, cavity design integrated with low‐dimensional emitters such as quantum dots, hBN, and TMDs is central to exploring magnetic properties in polaritons, referred to as magnons [31, 32]. Reviews of plasmon–polaritons in metals are generally restricted to metallic nanoparticles, without consideration of two‐dimensional forms, functional integration, or device relevance [33, 34, 35]. This underscores the need for a comprehensive review that spans fundamental physics to device‐level applications of polaritons in 2D materials, highlighting the importance of advanced characterization tools capable of probing these interactions with atomic‐scale resolution to identify bottlenecks in device performance.

**FIGURE 1:**
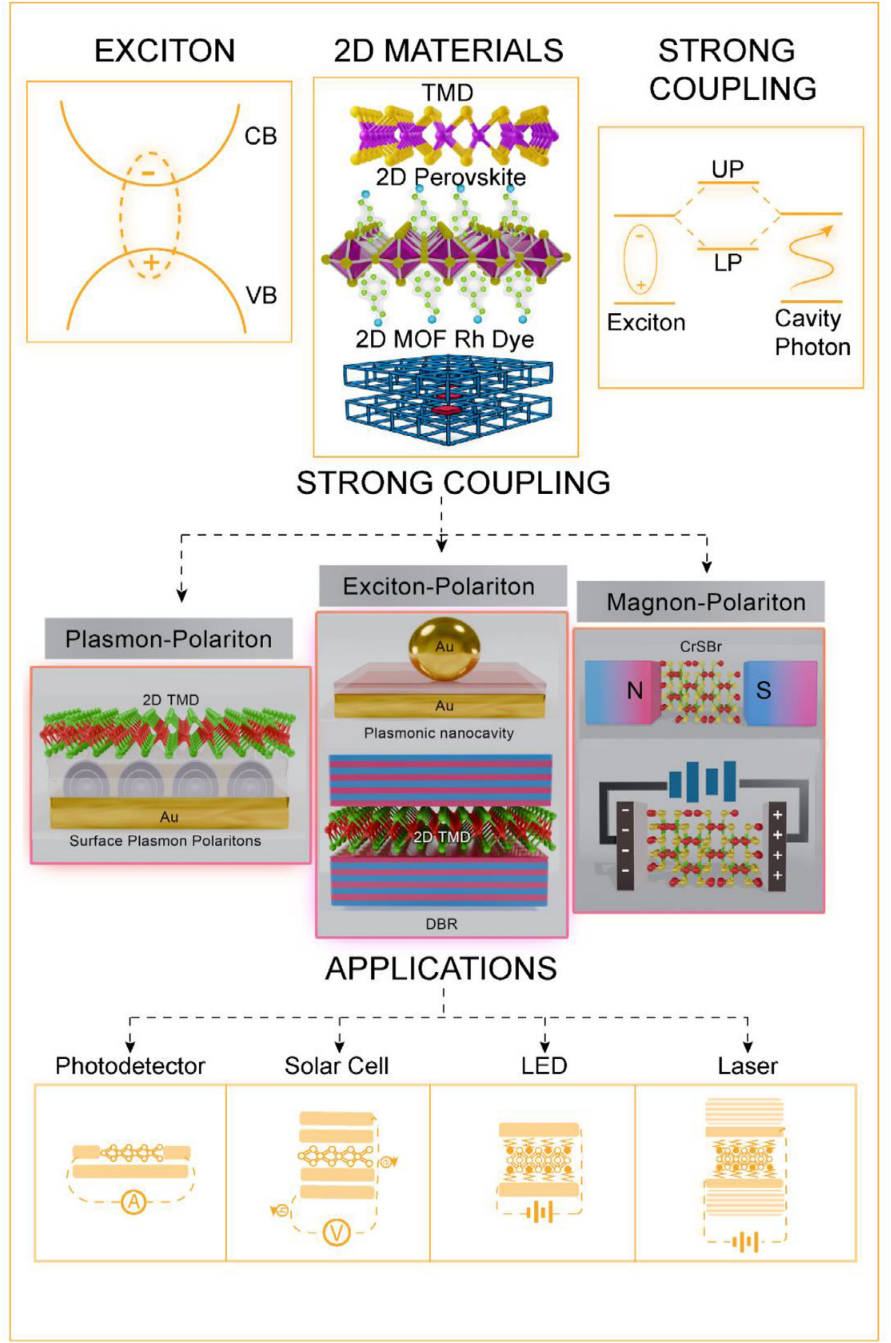
Exciton and strong coupling (exciton–polariton, plasmon–polariton, magnon–polariton) based optoelectronic devices are focused on in this review.

In this review, we present recent progress in 2D optoelectronic devices based on excitons and strong light–matter coupling (polaritons), emphasizing their unique advantages (Figure 1). We also discuss emerging polaritonic devices involving interactions between light and excitons, plasmons, or magnons, and identify key challenges hindering practical implementation. Strong coupling, while central to low‐threshold laser operation, also extends to applications in photodetectors and solar cells. The scalability and tunability of 2D optoelectronic properties make these systems attractive for space‐compatible solar cells [36, 37], chip‐integrated lasers [38], flexible display LEDs [39], and quantum detectors [40]. We have covered recent advancements on using strong coupling for photodetectors, LEDs, and on‐chip of these devices, which are less emphasized in the previous review. We not only summarize device demonstrations but also extract design rules for achieving high power density, low threshold, or spectral tunability under strong coupling. These devices can pave the way for all‐2D‐based integrated photonic circuits, flexible light emitters, and solar cells. Finally, we highlight the critical role of electron microscopy in probing strong light–matter interactions at atomic resolution. By outlining present challenges and future directions, this review provides a roadmap for next‐generation exciton‐ and polariton‐based technologies.

Before going into details of excitons and polaritonic systems, we would like to give a brief introduction to these quasiparticles. This familiarity with these quasiparticles will help the reader to understand the device aspect.

Excitons: Excitons are quasi‐particles made up of bound electrons and hole pairs. Excitons form via free carrier association, enabling luminescence in LEDs, and dissociation into unbound electrons and holes drives solar cells. In 2D materials such as TMDs, excitons can exhibit high binding energy in the order of hundreds of meV due to low dielectric screening. Depending on the material type, the binding energy of excitons varies and can be utilized in various device applications. Their formation pathways and dissociation/emission capabilities make excitons central to optoelectronic device technologies from LEDs to photovoltaics. The list of acronyms used in this review article are provided in Table 1.

**TABLE 1:** List of acronyms used in this review article.

Acronym	Full form
LED	Light‐emitting diode
TMD	Transition metal dichalcogenide
2D/1D/0D	Two‐dimensional/one‐dimensional/zero‐dimensional
CIGS	Copper Indium Gallium Selenide
PCE	Power conversion efficiency
CTL	Charge transport layer
ETL	Electron transport layer
HTL	Hole transport layer
hBN/cBN/rBN/bBN	Hexagonal boron nitride/ Cubic boron nitride/ Rombohedral boron nitride/ Bernal stacked boron nitride
MA/FA	Methylammonium/Formamidinium
UV	Ultraviolet
PEC	Photoelectrochemical
NEP	Noise equivalent power
LASER	Light amplification through stimulated emission of radiation
PLQY	Photoluminescence quantum yield
SOC	Spin–orbit coupling
CW	Continuous wave
QCW	Quasi‐continuous wave
ASE	Amplified spontaneous emission
DBR	Distributed Bragg reflectors
BIC	Bound states in the continuum
PeVCSELs	Perovskite vertical‐cavity surface‐emitting lasers
WGM	Whispering‐gallery mode
RP	Ruddlesden–Popper
	
MOF	Metal–organic framework
EL	Electroluminescence
BHJ	Bulk heterojunction
FHJ	Flat heterojunction
UPB	Upper polariton branches
LPB	Lower polariton branches
SWNT	Single wall nanotube
EQE/IQE	External Quantum Efficiency/ Internal Quantum Efficiency
TPP	Tamm plasmon polaritons
SPP	Surface plasmon polaritons
FWHM	Full‐width at half‐maximum
NIR	Near‐infrared
LiDAR	Light detection and ranging
ATR	Attenuated total reflection
EELS	Electron Energy Loss Spectroscopy
CL	Cathodoluminescence
LDOS	Local density of states
STEM	Scanning tunnelling electron microscope
CMOS	Complementary metal–oxide–semiconductor

Polaritons: Polaritons are a hybrid state of light and matter that forms in the presence of strong coupling. We will focus more on strong coupling in Section 3, where we will define all the important parameters for achieving polaritons and strong coupling.

In this review, we will focus our attention on the following questions and find promising strategies for developing better optoelectronic devices based on 2D materials:

1. Which class of semiconductor (2D, mixed‐dimension, or 3D) is best suited for photovoltaics, photodetectors, and lasing applications?
2. Which device design exploits the maximum performance from an exciton‐based system?
3. What device metrics benefit most from strong coupling?
4. Which 2D platforms are most promising for electrically driven polariton devices?

## 2 Exciton‐Based Optoelectronic Devices

### 2.1 Solar Cells

Solar cells convert sunlight into electricity by absorbing photons, generating excitons, and separating them into free carriers. Conventional devices based on monocrystalline silicon, CIGS, or CdTe achieve good performance; however, they are limited by high production costs and toxicity concerns. Perovskite solar cells, which are solution‐processable, offer a more cost‐effective alternative; however, they suffer from Pb toxicity and lower stability compared to silicon. In recent years, solar cells based on 2D materials have gained attention for both terrestrial and space applications. For space and floating solar technologies, stability and a high power‐to‐weight ratio are critical, and 2D materials excel in these aspects due to their strong light absorption and high charge mobility. In this section, we review advances in solar cells derived from 2D materials and mixed‐dimensional structures (2D/0D), and compare their efficiencies with perovskite solar cells, which have recently surpassed those of silicon.

#### 2.1.1 2D TMD‐Based Solar Cells

TMDs have drawn considerable interest for solar cell applications owing to their direct bandgap in the monolayer form and their high absorption coefficients compared to silicon, III–V semiconductors, and perovskites. For example, monolayer TMDs, particularly MoS_2_, exhibit stronger absorption than Si and GaAs, with a nanometer‐thick MoS_2_ layer achieving absorption comparable to a 50 nm Si layer and generating currents of up to 4.5 mA/cm^2^ [41]. Their efficient light absorption in ultrathin active layers (∼10 nm), combined with high charge‐carrier mobility and the possibility of large‐area growth, makes TMDs highly promising candidates for wafer‐scale solar cells with power densities exceeding 100 W/g. More detailed calculations on high‐power‐density solar cells can be found elsewhere [42].

**FIGURE 2:**
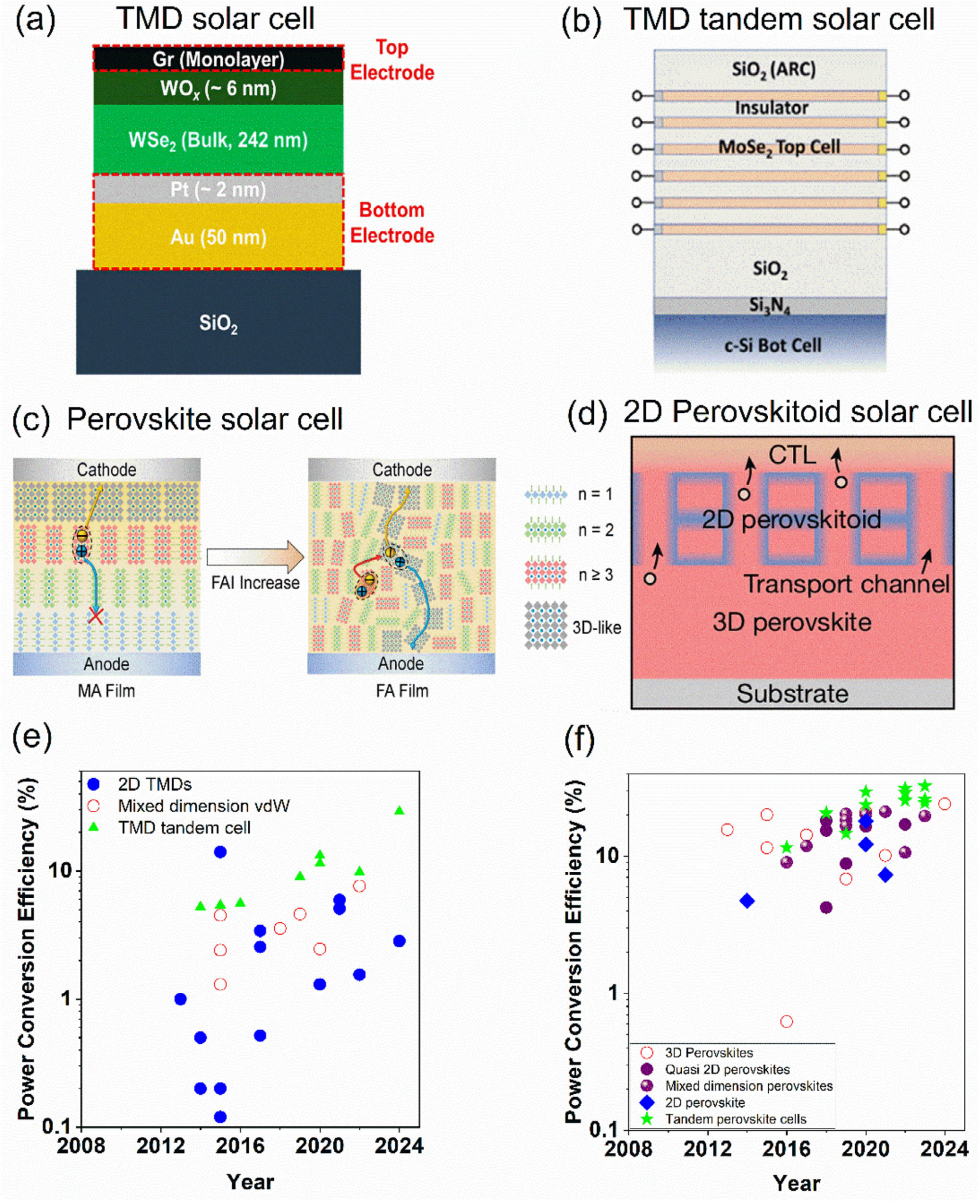
2D materials‐based solar cells and power conversion efficiency. (a) A WSe_2_‐based solar cell with PCE 5.44% (Reprinted/ adapted with permission [43] 2022, American Chemical Society), (b) A MoSe_2_‐based tandem solar cell (Reprinted/ adapted with permission [44] 2024, American Chemical Society). (c) schematics of an MA to FA‐based 2D perovskite solar cell, which shows enhanced PCE due to well‐connected 3D perovskites (Reprinted/ adapted with permission [45] 2021, Wiley‐VCH GmbH. (d) Illustration of the structural alignment of 2D perovskitoids on top of a 3D perovskite (Reprinted/ adapted with permission [46] 2024, Springer Nature). Power conversion efficiency from 2D materials‐based solar cells in (e) Monolayer, mixed‐dimension (2D/0D) and tandem cells from TMDs [41, 44, 47, 48, 49, 50, 51, 52, 53, 54, 55, 56, 57, 58, 59, 60, 61, 62, 63, 64, 65, 66, 67, 68, 69] and (f) 2D, quasi‐2D perovskites compared with 3D perovskites and tandem perovskites [45, 70, 71, 72, 73, 74, 75, 76, 77, 78, 79, 80, 81, 82, 83, 84, 85, 86, 87, 88, 89, 90, 91, 92, 93, 94, 95, 96, 97, 98, 99, 100, 101, 102, 103, 104, 105, 106, 107, 108].

Monolayer TMDs such as MoS_2_ and WS_2_ were among the first materials employed in Schottky barrier solar cells owing to their favorable optoelectronic properties. The earliest monolayer WSe_2_ p–n diode, created using electrostatic doping, achieved a modest power conversion efficiency (PCE) of ∼0.5%. Nevertheless, its power density far exceeded that of conventional Si and GaAs‐based solar cells [47]. The low PCE is primarily attributed to limited light absorption, which can be improved through optical engineering [48] or by increasing the TMD thickness. For example, a 25 nm WS_2_ layer in an ITO/WS_2_/Au device reached a PCE of 1.7% [109]. However, further increasing the thickness beyond 100 nm (from 110 to 220 nm) resulted in only a marginal rise in PCE, from 0.7% to 1.8% [110]. Theoretical studies suggest that a 50 nm TMD layer could achieve a PCE of ∼25% [111]. Incorporating a WO_x_ interlayer into multilayer TMD/graphene heterostructures has been shown to enhance carrier separation by strengthening the electric field and enabling selective electron transport. A 242 nm WSe_2_ layer paired with a WO_x_ electron‐selective contact delivered a record PCE of 5.54% for a Schottky barrier solar cell (Figure 2a) [43]. Similarly, depositing MoO_x_ on graphene/WSe_2_ (200 nm) produced a PCE of 5.5% on a flexible polyimide substrate [49]. Thus, the introduction of MoO_x_ or WO_x_ layers plays multiple roles: improving carrier separation, enhancing the electric field, enabling selective electron transport, and serving as an anti‐reflective coating. Using multilayered MoSe_2_ on hBN highest PCE of 14% was reported [62]. In this configuration, the electrostatic doping via two gate contacts with the MoSe_2_ layer creates a p–n junction within the MoSe_2_ layers. Further, the hBN layer provided electrical insulation, which helps in the separate control of the p–n junction by the top and bottom gate control. The built‐in electric field enables rapid charge separation and efficient current generation. Compared to other p–n junction‐based devices, this device offers the additional advantage of defect suppression, which is generally caused by chemical doping or heterojunction formation. Moreover, the device benefits from enhanced light absorption due to the multilayer MoSe_2_ stacked on hBN.

TMD heterostructures, whether vertical or lateral, show distinct performance characteristics. Vertical heterostructures such as MoS_2_/WSe_2_ and MoS_2_/MoTe_2_ yielded PCEs of 0.2% and 0.3%, respectively [50, 112]. Incorporating graphene as a charge‐collector layer in WSe_2_/MoS_2_ vertical heterostructures significantly improved carrier extraction, raising the PCE to 3.4% [48] and boosting collection efficiency to 70%. In contrast, lateral heterojunctions of WSe_2_/MoSe_2_ achieved a PCE of 0.12% under 514 nm illumination, while WSe_2_/MoS_2_ heterostructures reached 2.56% under white light [51]. In the WSe_2_/MoSe_2_ lateral heterojunction, the depletion region lies fully in the monolayer plane and is completely exposed to light, thereby maximizing the active area and carrier generation. The illumination angle also does not alter PCE significantly and drops only by 5% upon changing the incidence angle from 0 to 75^0^. Although experimental efficiencies remain modest, theoretical predictions suggest that optimized TMD heterostructures could reach PCEs of ∼20%, particularly for systems such as MoSe_2_/WS_2_, MoTe_2_/MoSe_2_, and MoTe_2_/WS_2_ [113].

A key limitation of TMD heterostructures is their restricted light absorption, especially when compared with mixed‐dimensional solar cells. Mixed‐dimensional architectures, which integrate materials of different dimensionalities (0D, 1D) with 2D TMDs, have shown consistently higher performance. For example, incorporating PbS quantum dots into a MoS_2_/WSe_2_ heterostructure increased the external quantum efficiency to 70% and the PCE to 7.65% [52]. The incorporation of PbS QDs on MoS_2_/WS_2_ heterostructure facilitates broadband absorption (405–1064 nm) in the device [52]. Further, the type‐II band alignment in the heterostructure creates a built‐in electric field, which rapidly separates photogenerated electron–hole pairs. This efficient charge separation, combined with selective carrier transport through MoS_2_/WSe_2_ layers, generates substantially higher photocurrent compared to heterostructures without PbS quantum dots. The synergistic coupling between quantum dot absorption and heterostructure band engineering for charge separation and extraction drives superior photovoltaic performance. These designs improve absorption, enable bandgap tuning, and enhance carrier collection relative to monolayer devices.

Tandem solar cells represent another promising direction. An Al/MoS_2_/p‐Si tandem structure achieved a PCE of 5.23%, a 12% improvement compared to Al/p‐Si devices, due to enhanced light absorption by MoS_2_ below 680 nm [61]. Increasing MoS_2_ thickness in a graphene/MoS_2_/p‐Si/Si/TiO_x_ device further improved absorption, resulting in a PCE of 13.18% [53]. The dielectric environment around the TMD layer also plays a critical role in terms of efficient charge extraction or light confinement. For instance, replacing Si with GaAs and inserting hBN interlayer between MoS_2_ monolayer and GaAs raised efficiency to 5.42% by improving the Schottky barrier and optimizing charge separation [54]. Similarly, exploiting a unilateral depletion mechanism in MoS_2_/AsP heterostructures increased PCE to 9% [55]. Besides exploring different heterostructures for a tandem device, enhancing carrier mobility in the 2D layer is also crucial: doping MoS_2_ with thiourea or applying electrostatic gating in multilayer MoS_2_/p‐Si devices improved PCE to 9.81% [56]. Overall, tandem solar cells based on TMDs currently achieve efficiencies of ∼10%. Simulation studies predict that TMD superlattice/Si tandems could reach 12.31% from the TMD and 18.51% from Si, for a combined PCE of ∼30.94% (Figure 2b) [44]. This MoSe_2_/Si tandem solar cell shows improved absorption through band engineering, with the TMD superlattice placed on top to absorb high‐energy photons, while the underlying silicon layer absorbs low‐energy photons. Absorption is further enhanced by exploiting the refractive index mismatch between TMD and the insulating spacer layer between the TMD layers. To achieve additional absorption enhancement, a bottom SiO_2_ layer and an anti‐reflection coating consisting of Al_2_O_3_ (top) and Si_3_N_4_ (bottom) were placed, maximizing the absorption efficiency of the designed tandem solar cell. Progress has also been made toward direct integration: using thermolytic deposition, MoS_2_ has been directly grown between amorphous and crystalline n‐type Si on a p‐type Si substrate, achieving a PCE of ∼11.47%[57]. Looking forward, theoretical models suggest that dual‐gate tandem structures such as WTe_2_/MoSe_2_ or few‐layer TMD/Si combinations could push efficiencies to ∼30.4% [44, 114].

#### 2.1.2 2D Perovskite‐Based Solar Cells

2D perovskites are strong contenders alongside TMDs for realizing high‐power‐density solar cells. Ruddlesden–Popper perovskites ((BA)_2_(MA)_3_Pb_4_I_13_) have achieved a PCE of ∼12.51% in a simple planar configuration, far outperforming spin‐coated counterparts, which typically yield only 3%–4%[78]. Substituting BA with methylammonium (MA)[79] or replacing MA with formamidinium (FA)[45] in (4FPEA)_2_(FA)_4_Pb_5_I_16_ further increased PCEs to 16.92% and 21%, respectively. These improvements are attributed to reduced exciton binding energies and an extended absorption edge up to 806 nm. In purely MA‐based 2D perovskites, gradient‐oriented growth with decreasing “n” values restricts charge extraction. By contrast, FA substitution promotes randomly oriented 2D perovskite domains interconnected with 3D phases, enhancing charge transport and boosting efficiency (Figure 2c). FA‐based 2D perovskite devices have demonstrated record efficiencies of 21% and retained 97% of their initial performance after 1500 h at 85°C. Consequently, 2D–3D hybrid systems, often referred to as “mixed‐dimension perovskites,” represent a promising route toward simultaneously achieving high PCE and long‐term stability.

However, perovskite tandem solar cells have also demonstrated remarkable PCE around 32.5% using a perovskite/Si tandem configuration [107]. In this architecture, silicon enables the absorption of low‐energy NIR photons, while the perovskite layer absorbs high‐energy photons in the blue region. This broad spectral coverage (300–1200 nm) significantly enhances the PCE. The stability of the perovskite layer was improved by employing a triple halide composition, in which Cl was present at a higher concentration than Br and I. Additionally, increasing the Cs ion while reducing the MA content was the major factor contributing to enhanced perovskite stability. Interface trap states were further passivated using piperazinium iodide instead of LiF. To further boost efficiency, the photogenerated current density in the silicon bottom cell was increased by adding a rear reflector with a dielectric buffer layer (RDBL). Using this strategy, a PCE of 32.5% was achieved. Recently, a new class of 2D perovskites, termed perovskitoids, featuring face‐sharing PbI octahedra. The chemical formula of 2D perovskitoids can be represented as A_8_Pb_7_I_22_, which is different from the typical ABX_3_ perovskite structure. A heterostructure from 2D perovskitoid and 3D perovskite (azetidinium lead iodide (AzPbI_3_)) has demonstrated 85% retention of PCE at 85°C after 1500 h. These materials not only enhance charge transport but also suppress ion migration, a primary source of device degradation (Figure 2d). As perovskitoid‐based solar cells are emerging materials, further study is required to validate the scalability and stability in various environmental conditions. Table 2 summarizes the device strategies for the best‐performing 2D‐based excitonic solar cells.

**TABLE 2:** Comparison of performance and device strategies used in exciton‐based solar cells.

Device category	Active material	Spectral window	PCE (%)	Device strategy	Reference
**2D TMDs**	MoSe_2_	532 nm	14	Lateral p–n junction with multilayered MoSe_2_ on hBN	[62]
**Mixed dimension vdW**	PbS/MoS_2_/WS_2_	405–1064 nm	7.65	Increase absorption and spectral range via PbS QD incorporation	[52]
**TMD tandem**	MoSe_2_	400–1000 nm	30.9	TMD‐cSi Tandem	[44]
**3D perovskite**	FAPbI_3_	300–850 nm	24.1	Interface passivation using sodium copper chlorophyllin	[108]
**Quasi 2D perovskite**	(3‐BBA)_2_(FA/MA)_n−1_PbI_3n+1_	300–800 nm	18.20	Hydrophobicity, high crystallinity, and order	[71]
**Mixed‐dimension perovskites**	(4FPEA)_2_(FA)_4_Pb_5_I_16_	300–850 nm	21.07	The presence of FA promotes the 3D phase, while MA promotes the 2D phase. 2D–3D intermixing significantly enhanced the light absorption at the near‐infrared region, and facilitates the charge dissociation and charge transport through the 3D perovskite connected network.	[45]
**2D perovskite**	(MTEA)_2_(MA)_4_Pb_5_I_16_ (*n* = 5)	350–850 nm	18.06	Enhancement in charge transport through the sulphur–sulphur interaction between two MTEA molecules	[106]
**2D perovskitoids/3D perovskite**	Chemical formula for 2D perovskitoid is (A6BfP)_8_Pb_7_I_22,_ here, A6BfP corresponds to N‐aminohexyl‐benz[f]‐phthalimide hydroiodide and 3D perovskite is azetidinium lead iodide (AzPbI_3_)	300 – 800 nm	24.6	The enhancement in device stability can be attributed to the suppression of cation migration in the 2D perovskitoid phase. Furthermore, charge transport is enhanced by the increased octahedral connectivity in the 3D perovskite phase.	[46]
**Tandem perovskite**	Cs_0.22_FA_0.78_Pb(I_0.85_Br_0.15_)_3_ + 5% MaPbCl_3_	300–1200 nm	32.5	Three major factors—crystal stability, band alignment, and surface passivation led to improved efficiency. For crystal stability, the amount of Cs and Cl ion were increased, while reducing the MA content. The V_oc_ increased for the triple halide composition. Band alignment improvement and surface passivation using piperazinium iodide instead of LiF further contributed to the enhanced PCE.	[107]

A comparison of PCEs from TMDs, 2D perovskites, and mixed‐dimensional van der Waals structures (Figure 2e,f) highlights current limitations and opportunities. Single‐junction TMD‐based devices still exhibit efficiencies below 15%. Enhancements in charge mobility, effective doping strategies for p‐ and n‐type conduction, and stronger charge generation are needed to improve their performance. Alternative concepts, such as bulk photovoltaics, could also help dissociate strongly bound excitons. For tandem devices, calculations predict that TMD superlattice/Si and 2D perovskite/Si architectures could reach PCEs of ∼30%, enabling high power density. Notably, flexible 2D perovskite devices have already achieved power densities of 44 W/g and been scaled to areas of 1 cm^2^ [115]. Despite these advances, the inherent lead‐free composition and superior stability of TMDs remain advantageous, making them attractive not only for space‐based, high‐power‐density applications but also for lightweight, flexible solar technologies.

Table 2 summarizes the highest efficiency for each device category and the underlying mechanism for the devices evaluated in Figure 2e,f. As observed from Table 2, in atomically thin TMD, reduced dielectric screening leads to strongly bound excitons with binding energies far exceeding thermal energy at room temperature. Using lateral heterojunction and tailored contacts to effectively dissociate excitons into free carriers leads to a power conversion efficiency (PCE) of 14%. Further, electrostatic doping also facilitates a p–n junction in the MoSe_2_ multilayer. Nevertheless, the absorption range of TMD is limited, which limits the efficiency of the 2D solar cells. Introducing mixed‐dimensional vdW, such as PbS quantum dots on MoS_2_/WS_2_ heterostructures, expands the spectral window to the infrared region (1064 nm). Finally, TMD/Si tandem solar cells hallmark an efficiency of 30.9%, which is the best reported value to date.

On the other hand, for perovskite solar cells, the design challenge shifts from exciton dissociation to managing the inherent trade‐off between perovskite stability and charge transport. The intercalating organic cations that provide remarkable environmental stability also act as potential barriers to interlayer charge motion. This has led to a paradigm shift toward mixed‐dimensional 2D/3D structures. Here, the 3D network provides a pathway for efficient charge transport, while the 2D components passivate interfaces and suppress ionic migration, thereby enhancing the device efficiency. Overall, we discussed the progress of 2D materials toward solar cell applications. It can be noted that both TMD‐based and 2D perovskite‐based solar cells demonstrate that mixed‐dimension semiconductors can be a promising approach for enhancing the efficiency of solar cells. Beyond solar cells, these 2D materials find applications in other light‐sensing devices spanning the ultraviolet (UV), visible, and infrared regions. The ability of 2D materials to absorb infrared light makes them particularly suitable for unique applications in conventional telecommunications and quantum communication. Likewise, photon sensing in the UV and visible regions enables the miniaturization of photodetectors using ultrathin flat lenses based on 2D materials. The next section focuses on 2D‐material‐based photodetectors, expanding the discussion toward these emerging technologies.

### 2.2 2D Photodetectors

Photodetectors convert photons into electrical signals and are designed to operate with high responsivity, fast response times, low voltage requirements, and sensitivity to low‐intensity light. Two‐dimensional (2D) materials such as TMDs, perovskites, graphene, hexagonal boron nitride (hBN), and gallium oxide (Ga_2_O_3_) have been extensively studied for photodetection applications. Among them, hBN and Ga_2_O_3_ are particularly attractive for deep‐UV detection, competing with traditional 3D semiconductors like ZnO. In contrast, TMDs and 2D perovskites are well‐suited for visible light detection, while graphene‐based structures show strong promise for the infrared range. Below, we highlight a couple of benchmark devices in each spectral window based on the figure of merit in Table 3.

**TABLE 3:** Benchmarking 2D materials‐based photodetectors within each spectral window and their figures of merit. For each spectral window, the two best devices are reported, one with high responsivity and another with a fast response time.

Spectral range	Semiconductor	Device structure	Wavelength (nm)	Mechanism	Responsivity (A/W)	Dark current (A)	Detectivity (Jones)	Response time—rise/fall (s)	Operating voltage (V)	Reference
**Deep UV (200 nm to 400 nm)**	β‐Ga_2_O_3_ flakes	(Cr/Au)/β‐Ga_2_O_3_ / MgO /(Cr/Au)	260	Photogating	2.40 × 10^7^	6.7 × 10^−12^	1.7 × 10^15^	6.38/—	V_DS_ = 5, V_GS_ = −20	[125]
	Amorphous‐Ga_2_O_3_	Al‐doped ZnO/a‐Ga_2_O_3_/Al‐doped ZnO	254	Photoconductive	∼ 0.7	2.84 × 10^−12^	4.84 × 10^14^	24 × 10^−6^ / 1.24 × 10^−3^	10	[141]
**Blue (380 nm to 500 nm)**	PbI_2_ nanosheet	Au / PbI_2_ /Au	450	Photoconductive	147.6	1.0 × 10^−11^	2.56 × 10^11^	∼18 × 10^−3^ / 22 × 10^−3^	5	[153]
	PbI_2_ single crystal	Au / PbI_2_ /Au	450	Photoconductive	0.18	0.56 × 10^−9^	3.23 × 10^11^	323 × 10^−6^ / 520 × 10^−6^	10	[154]
	AgSePh	Graphene/AgSePh	450	Photogating	∼100			50 × 10^−3^ /150 × 10^−3^ (to 50%)		[152]
**Green (500 nm to 625 nm)**	Pr: CdS	Au/Pr:CdS/Au	532	Photoconductive	2.71	∼9.4 × 10^−7^	6.9 × 10^11^	0.090 / 0.170	10	[155]
	PbI_2_ nanosheet	Au/PbI_2_/Au	530	Photoconductive	2.3	∼ 10^−12^	1.5 × 10^12^	—/700 × 10^−6^	20	[156]
**Red (625 nm to 750 nm)**	BiSeI single crystals	BiSeI	635	Photoconductive	3.2	∼24 × 10^−6^	7 × 10^10^	145 × 10^−3^ / 98 × 10^−3^	0.1	[134]
	Monolayer WSe_2_	Lateral Graphene/WSe_2_/ Graphene	750	Split gate	∼2 × 10^−3^ to 5 × 10^−3^	Near‐zero dark current	—	∼16 × 10^−12^	V_bias_=0, V_asym_=10	[131]
**Near Infrared (750 nm to 1650 nm)**	MoS_2_/ HfS	Au /MoS_2_/HfS/Au	980	Photoconductive	1500	Few nA	2 × 10^14^	60 × 10^−6^/71 × 10^−6^	−5	[157]
	Graphene / WSe_2_ / Graphene	Graphene /WSe_2_/ Graphene	759	Field effect	44 × 10^−3^	Near‐zero dark current	1 × 10^8^	5.5 × 10^12^	V_g_=30, V_b_=0	[158]

#### 2.2.1 Deep UV Photodetectors

Hexagonal boron nitride (hBN) stands out due to its wide bandgap (∼5.82 eV), high absorption, and ultrafast response time [116, 117]. Various polymorphs of boron nitride, including cubic boron nitride (cBN), exagonal boron nitride (hBN), rhombohedral boron nitride (rBN), and Bernal stacked boron nitride (bBN), have been investigated, with hBN showing enhanced responsivity through bandgap engineering, photogating, and substrate optimization [117, 118, 119, 120]. Preliminary studies using boron nitride nanosheets (BNNS) in a metal–semiconductor–metal device structure have shown deep UV (250 nm) photodetection at temperatures of more than 100°C. Al/BNNS/Mo shows a photoconduction behavior with a responsivity of 1.5 mA/W (at 254 nm) at −5 V [117]. Further, replacing Mo with Cu substrate and using few‐layer hBN nanosheets as a light‐absorbing layer, the responsivity was increased to 5.022 A/W (at 210 nm) for the same voltage [121]. Also, the device showed high external quantum efficiency (2945%) and high responsivity (6.1 × 10^12^ Jones) due to the formation of a Schottky junction at the metal–semiconductor interface. Reported responsivity values range from mA/W to several A/W, often requiring trade‐offs between sensitivity and response speed [121]. One of the limitations in using hBN for deep UV photodetection is its high temperature synthesis (> 1000°C). To date, the lowest synthesis temperature of 2D hBN flakes using inductively coupled plasma‐enhanced chemical vapor deposition is ∼400°C – 500°C [122], yet, higher than the thermal budget for BEOL integration. Therefore, identifying low‐temperature processing of 2D materials for deep UV photodetection or wide‐bandgap semiconductors needs further investigation.

**FIGURE 3:**
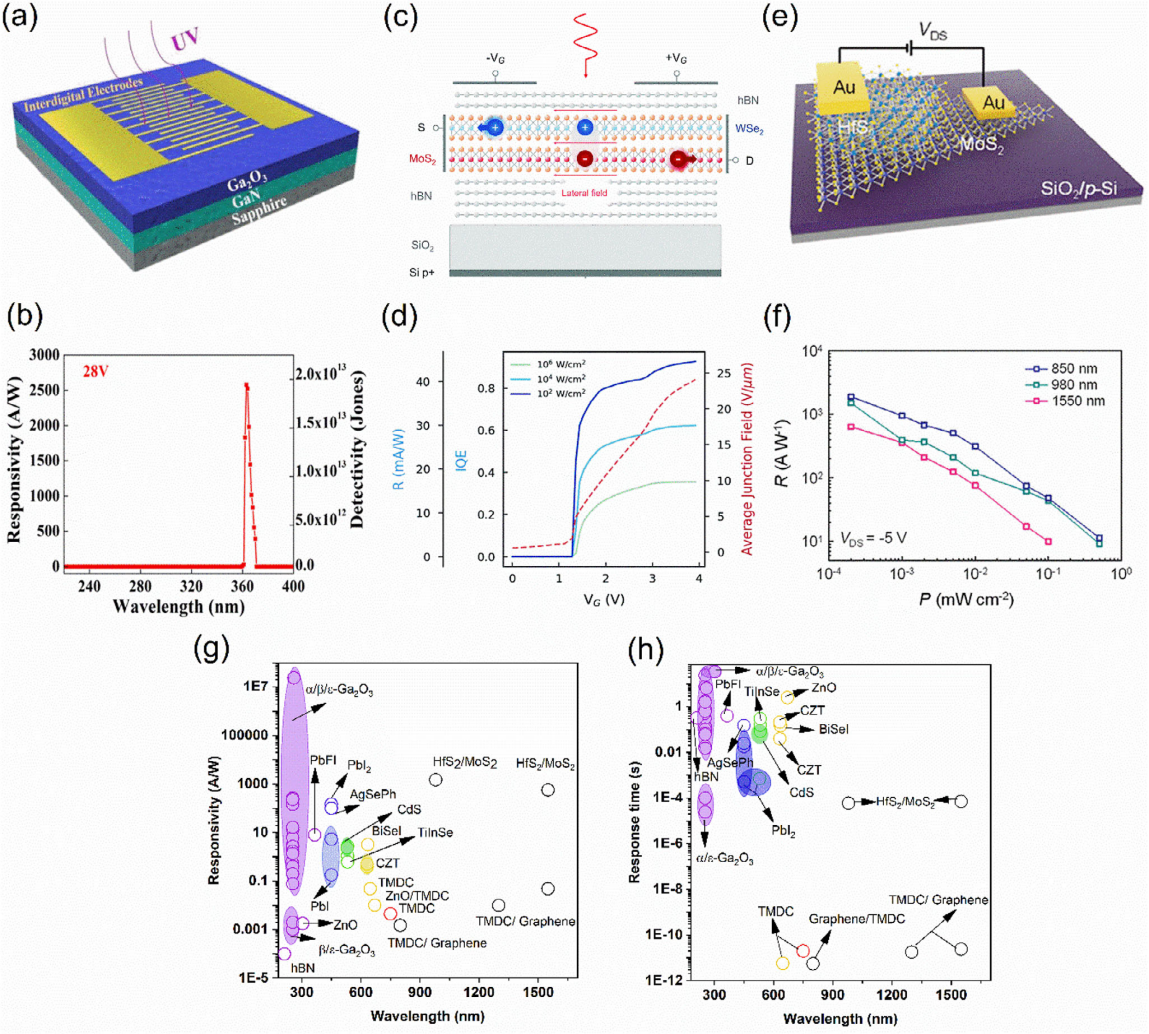
2D materials for UV to telecom region photodetection and Figure of Merits. (a,b) Schematic of the Ga_2_O_3_/GaN photodetector showing high responsivity (∼2580 A/W) at 28 V bias. Reproduced with permission. [123] 2022, American Chemical Society (c) Device structure of MoS_2_/WSe_2_ heterostructure for exploiting interlayer excitons in a split‐gate photodetector for ultrafast photodetection. (d) High responsivity at a split‐gate voltage (V_G_) >1 V with picosecond response time was observed. Reproduced with permission. [127] 2022, Royal Society of Chemistry (e) Schematic of HfS_2_/MoS_2_ photodetector; (f) Higher responsivity of the device at the infrared region as a function of power. Reproduced with permission. [128] 2024, Wiley‐VCH GmbH. (g) Responsivity and (h) response time of photodetectors using 2D materials (TMD, oxides, and 2D heterostructures) and 3D materials (oxides, chalcohalides, and PbI_2_). [123, 125, 127, 129, 130, 131, 132, 133, 134, 135, 136, 137, 138, 139, 140, 141, 142, 143, 144, 145, 146, 147, 148, 149, 150, 151, 152].

Recently, gallium oxide with a comparable bandgap [118, 123] was synthesized at relatively low temperature (150°C) using a liquid metal printing approach [124], showing excellent UV detection. This makes gallium oxide an alternative 2D material for deep UV photodetection. Gallium oxide shows several polymorphs, namely—stable β‐phase, metastable phases such as α‐Ga_2_O_3_ (trigonal), ε‐Ga_2_O_3_ (hexagonal), and γ‐Ga_2_O_3_(cubic), and unstable phase (δ‐ Ga_2_O_3_) [123]. Pristine Ga_2_O_3_ exhibits high responsivity (∼10^5^ A/W) at 260 nm [125], whereas in Ga_2_O_3_/GaN heterostructures (Figure 3a), the external bias voltage can change the maximum response wavelength due to increased charge depletion from the GaN layer at higher voltages. For instance, at low voltages (∼4 V), a narrow response at ∼256 nm was observed. Upon increasing the voltage to ∼12 V, broadband spectral response from ∼250 to 370 nm was observed. At 28 V, an ultranarrow band detection with exceptionally high responsivity (∼2580 A/W) and quantum efficiency around 363 nm was demonstrated, as illustrated in Figure 3b [123]. The ultranarrow‐band detectivity was due to the field‐enhanced exciton ionization process in the GaN layer, while the internal gain of the photodetector originated from the relatively large valence band offset between the Ga_2_O_3_ and GaN layers, which gave rise to high responsivity. In another study, using Ga_2_O_3_ / MgO heterostructure, the highest responsivity of 2.4 × 10^7^ A/W was observed via the photogating mechanism [125]. Though field effect transistors of Ga_2_O_3_ show a responsivity of 4.7 × 10^5^ A/W [126], the responsivity of Ga_2_O_3_ / MgO is further increased due to the suppressed back channel conduction (i.e., reducing electron hole pair recombination by transferring the photo electron to the MgO layer) via a defect‐assisted charge transfer process [125].

Furthermore, ε‐Ga_2_O_3_ exhibits ferroelectric properties that remain largely unexplored for photodetection applications. Recent advances in fabrication, such as pressure‐assisted printing, are being used to stabilize Ga_2_O_3_ polymorphs and enable scalable device production [124]. Overall, both hBN and Ga_2_O_3_ hold great promise for UV‐A and UV‐B photodetection, though significant opportunities remain for extending performance into deeper UV regimes.

#### 2.2.2 Red Photodetectors

Emerging chalcohalides such as BiSeI and Sn_2_SbS_2_I_3_ represent promising alternatives to lead‐based perovskites. Chalcohalide devices operate on the principle of photoelectrochemical (PEC) photodetection. The built‐in electric field formed at the electrolyte–semiconductor interface enables self‐powered operation [134]. BiSeI single crystals grown via physical vapor transport have demonstrated high responsivity (3.2 A/W), detectivity (7 × 10^10^ Jones), and external quantum efficiency of 622%. More detailed reviews of PEC‐based devices can be found elsewhere [159].

#### 2.2.3 Near Infrared Photodetectors

Photodetection in the visible to telecom region (∼1500 nm) relies heavily on TMDs, either as monolayers or heterostructures [160, 161, 162]. Vertical TMD p–n junctions exhibit high responsivity and fast response times due to the strong built‐in electric field in Type‐II heterojunctions. In contrast, lateral junctions achieve high optical gain through charge trapping, enabling avalanche photodetection. The simulated TMD hetero‐bilayers, such as WSe_2_/MoS_2_, encapsulated by hBN in a split‐gate configuration, have shown near‐zero dark current, picosecond response times, responsivities of ∼50 mA/W, and internal quantum efficiencies up to 95% (Figure 3c,d) [127, 132]. Use of a split gate electrode induced a lateral p–n junction and a very strong lateral electric field, which dissociates excitons faster and eventually generates photocurrent. These performance metrics are primarily enabled by efficient interlayer exciton dissociation. Substituting with HfS_2_/MoS_2_ hetero‐bilayers further extends detection into the infrared, achieving responsivities of ∼600 A W^−1^, detectivity of ∼7 × 10^13^ Jones at 1550 nm, and *R* of ∼1500 A W^−1^, detectivity of ∼2 × 10^14^ Jones at 980 nm, along with microsecond response times significantly faster than MoS_2_‐based devices [128]. Such heterostructures are particularly promising for single‐photon detection in the telecom regime with appropriate device engineering.

As shown in Figure 3g,h, the performance of 2D photodetectors is governed by fundamental trade‐offs between responsivity and response time. This is vividly illustrated within TMD heterostructures where, theoretically, WSe_2_/MoS_2_ p–n junctions achieve ∼4 ps response times and self‐powered operation at ∼50 mA/W responsivity through efficient interlayer exciton dissociation [127], while HfS_2_/MoS_2_ photoconductive devices attain >2500 A/W responsivity, however, require microsecond response times and external bias [128]. Similar compromises appear across the spectrum, where in deep UV, Ga_2_O_3_/GaN heterostructures achieve ∼2580 A/W responsivity at the cost of response times of several seconds [123, 136, 137, 138, 139]; Likewise, in the near‐IR, graphene/MoTe_2_ waveguide‐integrated detectors achieve bandwidths up to 46 GHz with responsivities in the tens of mA/W [132]. These trends are explicitly observed in Table 3 between two devices in each spectrum and highlight the inherent gain–bandwidth trade‐off. Similarly, trap‐assisted gain vs. noise trade‐off exists. The G‐r noise is due to stochastic generation and recombination of carriers from deep defect states, increases with the photoconductive gain, and is dominant in photoconductive‐based devices [163].

Gain mechanisms boost responsivity; however, they increase response time, whereas junction‐based designs prioritize fast response through rapid carrier extraction. Emerging approaches like hot‐carrier transfer in type‐I TMD heterojunctions with femtosecond response times [164] can help in circumventing classical limits; however, they face challenges in responsivity and scalability. Consequently, the architecture defines the performance trade‐off, where junction‐based devices use built‐in fields for fast, high‐responsivity operation, whereas photoconductive designs exploit long carrier lifetimes for high gain at the expense of response time. On the other hand, photo‐gating‐based designs facilitate additional control and improve responsivity [165].

The preceding sections examined the conversion of incident photons into excitons and their dissociation into free charge carriers, with device performance quantified by power conversion efficiency in solar cells and responsivity in photodetectors. Table 3 summarizes state‐of‐the‐art photodetectors operating in the deep‐UV, blue, green, red, and infrared spectral regions. Conceptually, the same device architectures can be employed in reverse, wherein injected charge carriers recombine radiatively to produce light, giving rise to light‐emitting diodes (LEDs). High‐purity, spectrally narrow emission from such devices underpins emerging applications in photonic integrated circuits and advanced display technologies. Accordingly, the next section reviews recent progress in 2D‐material‐based LEDs, emphasizing devices with high emission efficiency and low turn‐on voltage, and explores structural commonalities with high‐performance photodetectors to distill general design principles.

### 2.3 2D Light‐Emitting Diodes

For high‐performance and bright LEDs based on 2D materials, two essential requirements must be satisfied: strong intrinsic luminescence and efficient charge‐carrier injection into the active layer [166]. The photoluminescence quantum yield (PLQY) determines radiative exciton emission, whereas non‐radiative pathways such as the Shockley–Read–Hall process (where conduction electrons can relax to the defect level and then annihilating with a hole), Auger recombination (where the electron in the conduction band recombines with the hole in valance band by transferring its energy to another electron which later thermalizes and produces lattice heat), and defect‐assisted decay reduce device efficiency [167]. In addition to the optical quality of TMDs, efficient charge injection requires metal contacts that ideally form Ohmic junctions, thereby minimizing resistive losses and enabling effective carrier transport [168]. In this section, we focus on the optical aspects of 2D‐material LEDs, while readers are referred elsewhere for a detailed discussion of electrical considerations. We specifically highlight progress reported after 2023. Recent developments include polarized TMD‐based LEDs [166, 169], near‐infrared emission from 2D heterostructure LEDs [170], and the evolution from on‐chip unpolarized emission to devices capable of producing linearly and circularly polarized light.

**FIGURE 4:**
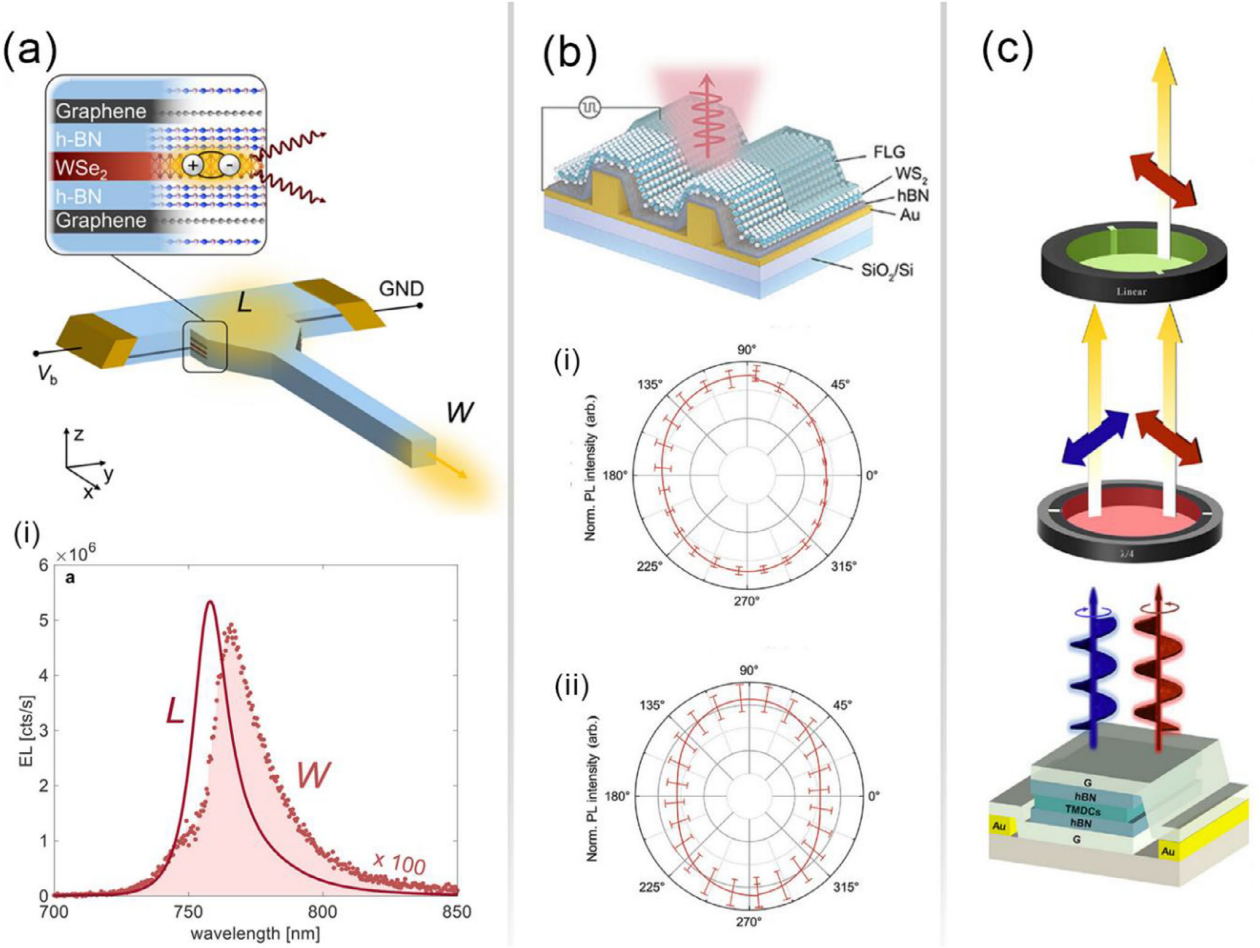
2D Exciton‐based LEDs. (a) Illustration of hBN waveguide coupled LED with WSe_2_ as active medium and graphene as electrode for charge injection (i) EL spectra at location L and W marked in the above panel (Reprinted/ adapted with permission [171] 2023, American Chemical Society (b) a schematic of on‐chip device fabrication with a monolayer of TMD demonstrating linear‐polarized EL for (i) WS_2_ and (ii) WSe_2_ (Reprinted/ adapted with permission [174] 2024, Wiley‐VCH GmbH. (c) Schematics showing circular polarized light measurement using a quarter‐wave plate and linear polarization setup using a 2D TMD encapsulated with hBN and graphene layer (Reprinted/ adapted with permission [175] 2025, Wiley‐VCH GmbH.

A particularly promising advancement is the integration of 2D LEDs with waveguides, a step forward for photonic integrated circuits. In one example, a WSe_2_ monolayer encapsulated between hBN layers was patterned to form the waveguide structure [171]. As illustrated in Figure 4a, the thicknesses of the top and bottom hBN layers were optimized to position the WSe_2_ monolayer at the peak of the optical field within the waveguide mode. This overlapping of modes optimized the coupling efficiency and helped the photons to come out efficiently instead of being lost due to reabsorption and scattering outside the mode. The device achieved a maximum external quantum efficiency (EQE) of 4% at a bias of 1.7 V, outperforming MoTe_2_‐based integrated LEDs (0.5%) [172] and WSe_2_/CdS devices (0.2%) [173]. Figure 4a(i) shows the electroluminescence (EL) recorded at positions “L” and “W” along the waveguide. The observed red shift in the spectrum at “W” compared to “L” indicates light scattering within the waveguide.

This type of emerging 2D material‐based light integration, combined with the waveguide, represents a significant step toward photonic on‐chip integration. When WSe_2_ is electrically biased, the generated excitons emit photons that are guided by the hBN encapsulation. hBN is employed due to its high refractive index, ease of integrating 2D materials, and low optical loss. The WSe_2_ layer must be positioned such that it overlaps with the hBN mode to enable efficient propagation. During fabrication, the coverage of graphene and WSe2 should be well aligned; otherwise, a peak shift at location W, Figure 4a(i), may happen due to resonant absorption. These findings highlight the potential of integrated 2D LEDs, though further refinement of design parameters is required to optimize performance.

Beyond integrating 2D LEDs with waveguides for photonic circuits, achieving polarized electroluminescence represents the next milestone toward electrically driven lasers on waveguides. Polarized emission from 2D semiconductors is particularly important for applications in encryption and display technologies. Pristine monolayer WS_2_ and WSe_2_ typically exhibit unpolarized EL. However, by exploiting the flexoelectric effect through strain engineering, linearly polarized emissions with degrees of polarization of 11.6% and 17.4% have been demonstrated (Figure 4b). Figure 4b(i, ii) shows the linearly polarized PL intensity from WS_2_ and WSe_2_‐based devices [174]. The nonuniform strain induced by a pre‐patterned umbilicate back electrode produces asymmetric atomic displacements, leading to dipole formation and exciton anisotropy. As a result, emission is enhanced along the zigzag direction compared to the armchair direction. Importantly, the polarization remains perpendicular to the applied strain, regardless of crystal orientation or excitation.

Here, we discussed waveguide‐integrated and polarization‐controlled LEDs and explored the mechanism of these devices. These fabrication strategies can not only show directional and polarized emission, but also serve as a natural platform for exciton–polariton or spin–polariton devices because of their inherent optical confinement, electrical pumping capability, and direct control over light polarization. The waveguide can provide strong coupling platforms with significant advantages over traditional planar microcavities. A ZnO [176] waveguide‐based edge‐emitting exciton–polariton laser has shown a low threshold for emission compared to conventional planar cavities due to polariton condensation. A more detailed insight into polariton condensation and strong coupling will be provided in the next section.

These LED platforms can also revolutionize the spin–polariton‐based device by leveraging magnetic proximity effects from antiferromagnetic materials like CrI_3_ [177]. The spin‐polarized carrier tunneling has been demonstrated using a stack of WSe_2_ on top of CrI_3_ with a separation layer of hBN. The injected carrier in the emissive layer is preferentially aligned by the magnetic moment of CrI_3_ and, due to the valley spin coupling in WSe_2,_ automatically converts the spin information into circular polarized light with high efficiency. This device also showed the first demonstration of the chirality tuning (light‐handedness) with gate voltage in 2D materials without using external magnets.

Another breakthrough is the realization of circularly polarized electroluminescence in 2D TMDs. At cryogenic temperatures (2 K), valley‐polarized EL reversal from right‐ to left‐circular polarization was observed by tuning the magnetic field from –7 T to +7 T (Figure 4c). The applied magnetic field lifts valley degeneracy at distinct points (K, K’) in momentum space, resulting in preferential charge injection that breaks time‐reversal symmetry and generates EL with left‐ and right‐circularly polarized light [175]. W‐based TMDs and hetero‐bilayers such as WSe_2_/WS_2_ displayed strong polarization reversal, owing to the larger spin–orbit coupling (SOC) and dark exciton ground state of WS_2_. These features enable more effective magnetic control of polarization compared to MoS_2_, which has a smaller SOC and a bright exciton ground state. Unlike chiral light emission in 2D perovskites, which arises from chiral amine groups, TMDs lack such intrinsic mechanisms due to their strong in‐plane excitonic behavior. Superlattices, metasurfaces, or cavity‐confined TMDs may provide viable pathways to realize chiral emission in these systems [178, 179, 180]. A few other recent works have also demonstrated the chiral emission using a layer of achiral perovskite confined between a metal and a metasurface created by an array of triangular silicon particles with broken inversion symmetry [181].

In the previous section, we discussed the recent developments on LEDs, such as LED coupled to waveguides and their integration on‐chip for polarized emission. These types of devices increased the light extraction efficiency and directionality in the LEDs. However, the increased extraction efficiency and directionality will not be sufficient to meet the requirements for long‐range applications such as telecommunication and LiDAR. Moreover, the relatively larger spectral bandwidth (20–50 nm) and low coherence length (∼µm) for LEDs make them unfavorable for these applications. For these specific applications, we need a light source that can provide a very narrow spectral bandwidth (∼ 1 nm) with a long coherence length (∼ km). To fulfil these requirements, researchers use LASERs—Light Amplification by Stimulated Emission of Radiation. In the next section, we will discuss the progress on 2D materials for lasing applications and the device aspect of these exciton‐based lasers developed from 2D materials.

### 2.4 Excitonic Lasers

Excitons, bound electron–hole pairs formed upon light absorption, are central to lasing in 2D materials. Population inversion arises when electrons are pumped from the valence band into the conduction band and relax to their lowest energy states, producing a three‐level lasing system. Owing to their long exciton lifetimes and large binding energies, 2D materials are highly promising as optical gain media [182]. The performance of lasers is typically evaluated by emission linewidth, operating temperature, and lasing threshold under different excitation regimes, including nanosecond pulsed, quasi‐continuous wave (QCW), and continuous wave (CW) pumping. Among these, CW lasing with narrow linewidths (<1 nm) at room temperature is most desirable for electrically driven devices. Achieving this requires efficient radiative recombination and effective thermal management, often assessed through amplified spontaneous emission (ASE) studies.

**FIGURE 5:**
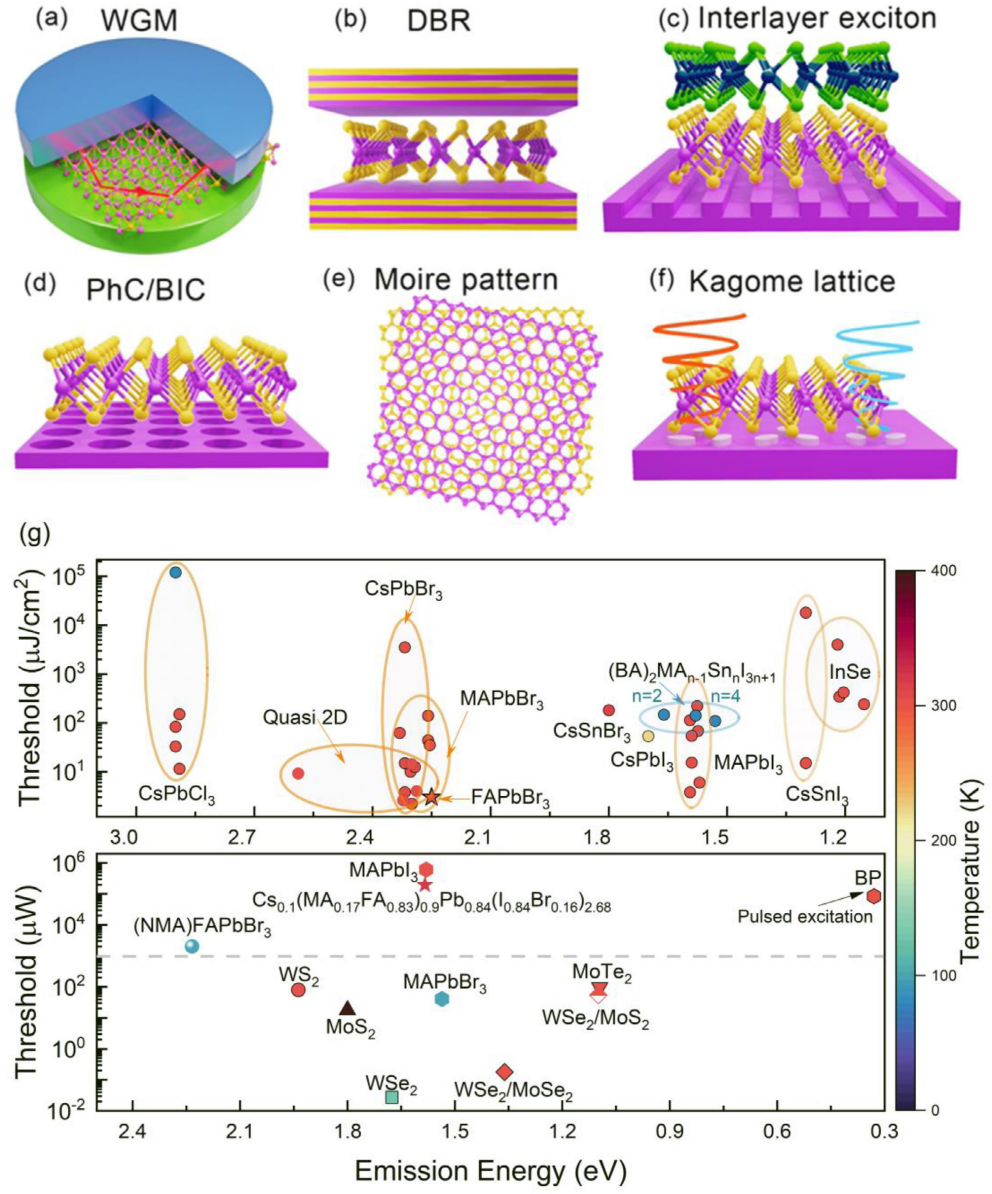
Different cavities for 2D excitonic lasers (a) A schematic for a TMDs microdisk laser using whispering gallery mode (WGM). (b) Lasing from a distributed Bragg reflector (DBR) using a 1L TMD as an active medium in which the intralayer exciton predominantly contributes to lasing. (c) Interlayer exciton lasing due to the stacking of two 1L different TMDs according to the resonance mode in the grating. (d) Lasing because of the photonic crystal structure resonance and the gain medium TMDs. Bound state in continuum ensures a high q‐factor for the cavity, enhancing the lasing (e). Twisted stacking of monolayers of TMDs leads toward localized period potential entrapping the exciton, which leads toward lasing (f). The image shows the spin valley Rashba monolayer laser. The structural configuration enables the generation of spin‐polarized light, where right‐handed and left‐handed circularly polarized modes emerge due to photonic Rashba splitting. The TMDs monolayer, embedded within the cavity, interacts with these spin‐polarized photonic modes, selectively exciting valley excitons. This process facilitates the formation of valley‐specific, spin‐polarized lasing from TMDs. (g) Optical pumping threshold and emission with respect to temperature [192, 193, 194, 195, 196, 197, 198, 199, 200, 201, 202, 203, 204, 205, 206, 207, 208, 209, 210, 211, 212, 213, 214, 215, 216, 217, 218] for 2D materials‐based excitonic lasers.

Lasing from monolayer TMDs and 2D perovskites has been demonstrated under both pulsed and CW excitation, though primarily at cryogenic temperatures [183, 184]. Figure 5a–d illustrates representative cavity designs, including whispering‐gallery modes, distributed Bragg reflectors (DBR), photonic crystals, and bound states in the continuum (BIC), localized, non‐radiating modes that confine light with exceptional efficiency [185, 186]. However, monolayer gain media remain constrained by limited tunability and challenges in junction formation. To address this, stacked 2D layers can create hybrid cavities that support lasing with high β‐factors. Furthermore, TMD heterostructures can host interlayer excitons [187], which exhibit longer lifetimes, tunable bandgaps, and high spatial coherence. In such systems, Moiré patterns act as exciton traps, suppressing recombination and facilitating lasing (Figure 5e) [188].

Beyond material optimization, substrate engineering provides additional routes to novel lasing phenomena. For instance, a WS_2_ monolayer integrated into a photonic microcavity with an inversion‐asymmetric Kagome lattice enabled a room‐temperature spin–valley Rashba laser (Figure 5f) [189]. Meanwhile, 2D γ‐InSe has emerged as a promising platform for on‐chip lasers in optical communication, demonstrating low‐loss, room‐temperature lasing near 1030 nm, an attractive regime for integrated optical gain media and cavity applications [190, 191].

Beyond TMDs, hybrid organic–inorganic metal halide perovskites have also emerged as promising lasing materials owing to their low‐cost processability, tunable bandgaps, and high photoluminescence quantum efficiencies of up to 70%[27, 219, 220]. Using 3D perovskites, a variety of devices, including vertical‐cavity surface‐emitting lasers (VCSELs) [221], distributed feedback (DFB) lasers [222], and whispering‐gallery mode (WGM) lasers [223] have been extensively explored [27]. In the search for lead‐free alternatives, tin‐based 2D halide perovskites, particularly Ruddlesden–Popper (RP) structures of the form (BA)_2_MA_n–1_Sn_n_I_3n+1_ (n = 1–4), have shown encouraging lasing behavior. However, the tendency of Sn^2+^ to oxidize into Sn^4+^ increases defect density and degrades lasing performance. Stabilization strategies, such as incorporating electron‐donating molecules, have been developed to mitigate this issue and make tin‐based perovskites viable candidates [192, 224].

A comparison of lasing thresholds for TMDs and perovskites under both continuous‐wave (CW) and pulsed excitation is presented in Figure 5g. Achieving room‐temperature CW lasing remains a central challenge. Among TMDs, MoTe_2_ has demonstrated CW lasing at room temperature [225], while WSe_2_ lasers are limited to operation below 80 K [184]. Except for black phosphorus, most TMD systems exhibit lower lasing thresholds under CW excitation compared to perovskites (see Figure 5g, bottom panel, dotted line). Interlayer excitons in TMD heterostructures further extend emission into the telecommunication range. InSe also supports infrared emission; however, it suffers from phase‐purity issues that hinder lasing [226]. Although blue lasing from TMDs has not yet been achieved, emerging semiconductors such as metal–organic chalcogenolates have demonstrated photoluminescence with narrow linewidths (∼1 nm), suggesting potential in this spectral region [227].

By contrast, perovskites have yet to achieve infrared lasing, likely due to the influence of Pb on their band structure. Yb^3+^‐doped CsPbCl_3_ exhibits infrared emission via quantum cutting; however, lasing has not been realized [228]. Attempts to substitute Pb with Sn for bandgap tuning remain limited by material instability. Room‐temperature CW excitonic lasing in perovskites is still elusive, although several systems have shown CW amplified spontaneous emission (ASE) or short‐duration CW lasing. Overall, perovskites generally exhibit higher lasing thresholds than TMDs, possibly due to lower defect tolerance. Nevertheless, exciton–polariton lasers offer a promising path toward room‐temperature CW operation in perovskites, representing an exciting future direction for the field [229].

As discussed from the outset, conventional excitonic lasing relies on population inversion, with lasing occurring only when the exciton density exceeds the Mott transition threshold. Under such high‐pump conditions, the resulting thermal load becomes a major limiting factor for exciton‐based lasers. This limitation motivates the development of alternative lasing schemes that can operate below the Mott density and therefore require substantially lower excitation levels. In this context, polaritonic lasing emerges as a promising alternative, as it does not strictly require population inversion. Instead, polaritons condense into the lower polariton branch, leading to coherent emission. This distinction defines the fundamental difference between excitonic and polaritonic lasers.

Nevertheless, excitonic gain remains advantageous at elevated temperatures and under off‐resonant excitation conditions, where strong coupling and polariton‐mediated processes are significantly weakened. In contrast, polariton lasing, which exploits strong exciton–photon coupling, enables low‐threshold operation and offers additional functionality through modulation via spin or valley degrees of freedom.

## 3 Strong Light–Matter Interactions for Optoelectronic Devices

In the preceding section, we reviewed excitonic devices and highlighted recent advancements in their performance. We now turn our attention to strong light–matter interactions and their various manifestations, to understand how these phenomena can introduce novel functionalities into excitonic devices. Specifically, we focus on three representative types of strong coupling: (1) exciton–polaritons, arising from strong interactions between light and matter; (2) plasmon–polaritons, involving the coupling of light with plasmons; and (3) magnon–polaritons, resulting from the interaction of light with magnons.

Before diving into polariton physics, it is necessary to understand some of the fundamental concepts. Upon exciting a semiconductor (exciton binding energy > kT at room temperature) placed in a resonating cavity, the interaction of excitons and photons leads to the generation of a half‐light, half‐matter quasi‐particle called a polariton. Some criteria should be followed for the effective generation of new particles. The energy of the exciton and the photons should match such that effective exchange occurs. The efficiency of coherent energy exchange between the cavity photon and the exciton is quantified by the term coupling strength (g). For effective coupling, the quality factor (Q‐factors) of the cavity should be high. Q‐factor is a dimensionless quantity that defines how effectively the electromagnetic wave resonates in the cavity. It is calculated by the ratio of resonating wavelength to its full‐width at half‐maximum. If the coherent energy exchange between photon and exciton is faster than dissipation, the regime is called strong coupling, in converse it is called weak coupling. A mathematically strong coupling regime is defined by

$$2g>\left(\frac{\gamma c+\gamma x}{2}\right)$$

where γc and γx are the rates of decay of photon and exciton, respectively, in the cavity.

In the regime of strong coupling, the resonance separates into two modes (eigenstates). These are called upper polaritons (UP) and lower polaritons (LP). The new energy states thus generated will be above and below the energy state of the exciton or photon energy level. The splitting of the exciton energy state into these two states is called Rabi splitting. Energy difference between the states at momentum k|| = 0 is Rabi splitting energy (ħΩ). Under weak coupling, the excitonic resonance broadens and enhances due to a higher number of optical states (Purcell effect).

### 3.1 Exciton–Polariton‐Based Optoelectronic Devices

#### 3.1.1 Exciton–Polariton‐Based Lasers

Polariton lasers operate through Bose–Einstein condensation of exciton–polaritons (E–Ps), where particles collectively occupy a low‐energy state, enabling lasing at ultrafast timescales and low thresholds. These properties make them highly attractive for low‐power optoelectronic applications. Progress in polariton lasing spans materials from 3D to 0D systems. III–V semiconductors such as GaAs [230] and GaN [231], II–VI compounds like ZnO [232], and organic materials [233, 234] have all demonstrated efficient lasing. In two‐dimensional systems, lead halide perovskites exhibit tunable polariton condensation [235, 236, 237] and integration into photonic platforms [238], while TMDs such as MoSe_2_ and WS_2_ have enabled bosonic condensation [239] and spin–polariton lasing [240] at cryogenic temperatures. More recently, 2D metal–organic frameworks have shown ultrastrong coupling with record‐low thresholds [241].

#### 3.1.2 Optically and Electrically Pumped Exciton–Polariton Lasers

**FIGURE 6:**
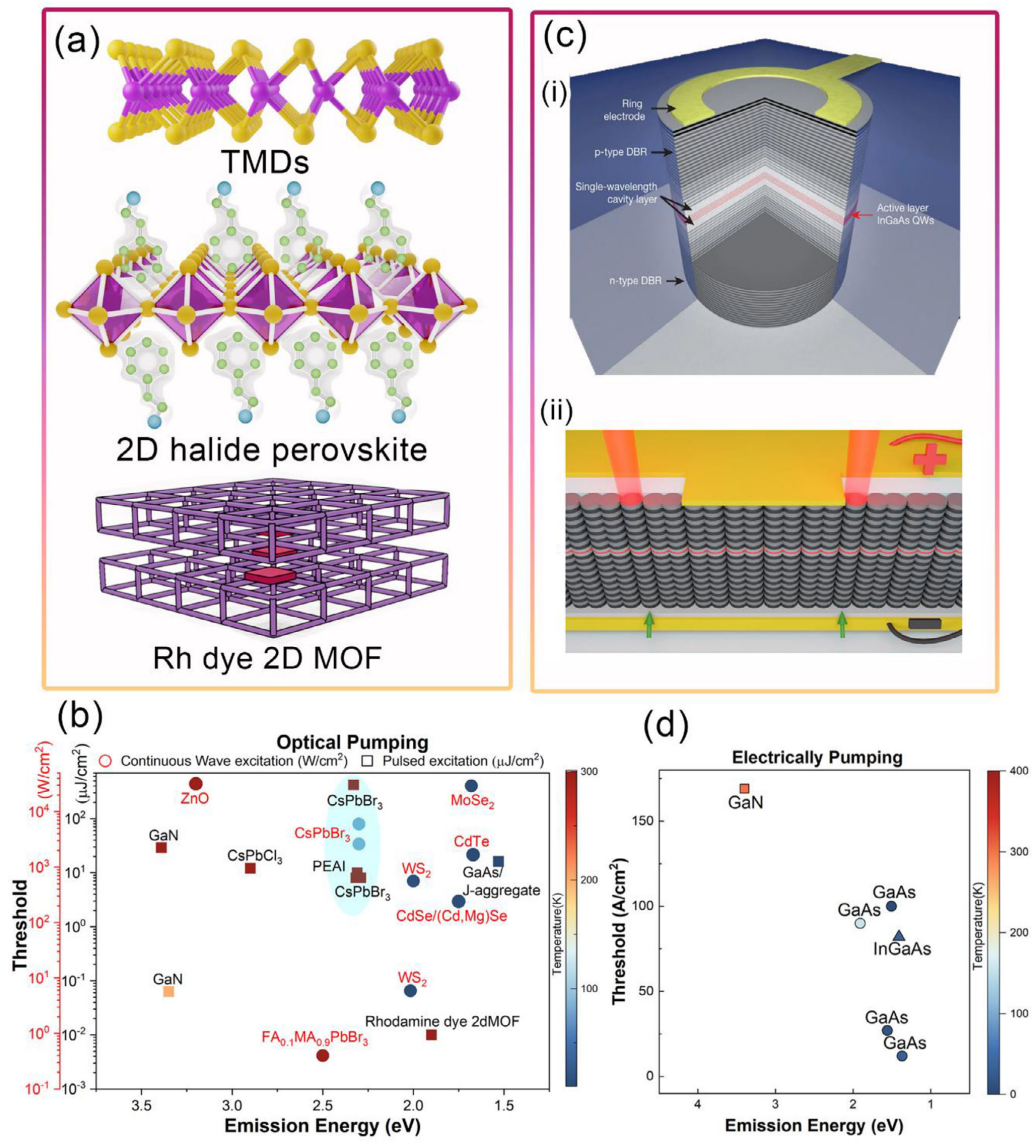
Optical‐pumped and Electrically‐pumped Polariton Lasers. (a) Emerging polariton lasers from 2D materials such as halide perovskite, TMD, and 2D MOF with Rhodamine dye (b) Comparison of the lasing threshold from various semiconductors using optical excitation (both CW and pulsed). The color of the symbol denotes the operating temperature, which can be referred to from the color bar. The red and black axis denotes the threshold for CW and pulsed excitation, respectively. The lasing threshold for various semiconductor materials is taken from the following references for GaN [231, 246] Perovskites [229, 235, 247, 248, 249, 250, 251, 252] TMDs [239, 243, 253] GaAs/J‐aggregate [254] Rhodamine dye 2D MOF [241] CdTe [255] ZnO [256] CdSe/(Cd, Mg)Se [257] (c) (i) Electrically pumped polariton laser having InGaAs quantum well in GaAs λ‐cavity. Reproduced under terms of the CC‐BY license [258] 2013, Zhang et al., Springer Nature (ii) An electrically pumped topological polariton laser which makes use of topological Su–Schrieffer–Heeger defect for the generation of lasing action. Reproduced with permission [245] 2024, American Chemical Society (d) Electrically pumped polariton lasing threshold from various materials and its operating temperature: GaN [259], GaAs [260, 261, 262], InGaAs [258].

Figure 6a,b summarizes the 2D materials developed for exciton–polariton lasers and the reported E–P lasing thresholds under continuous‐wave (CW) and pulsed excitation. In TMDs, lasing is typically achieved using CW excitation at cryogenic temperatures, while room‐temperature operation remains challenging due to defect‐induced exciton dissociation [242]. WS_2_, however, demonstrates an exceptionally low pumping threshold of 59 nW/µm^2^, attributed to its quantum‐well‐like structure with hBN spacers. A transition from single‐mode to multimode lasing has been observed as the temperature increases from 4 to 200 K [243]. Despite these advances, TMD‐based lasers are still limited by emission linewidths above 1 nm, which is higher than the ideal standard.

In contrast, perovskite‐based exciton‐polariton lasing is most often observed under pulsed excitation. A rare exception is FA_0.1_MA_0.9_PbBr_3_, which has shown lasing under low‐power CW pumping [244]. The blue‐shaded region in Figure 6b highlights perovskite‐based exciton‐polariton lasing. Notably, (PEAI)PbI_4_ exhibits biexciton photon lasing (50 µJ/cm^2^) at a threshold significantly lower than that required for exciton‐polariton condensation (200 µJ/cm^2^). This underscores the need for further investigation into perovskite systems to realize reliable low‐threshold operation.

Looking beyond optically pumped systems, achieving electrically pumped lasing remains a critical goal for device integration. At present, electrically pumped exciton‐polariton lasers have only been realized in 3D bulk semiconductors. The first simulation of an electrically injected polariton laser, reported in 2009, employed a bulk GaN microcavity. Figure 6c(i) depicts the first experimental demonstration of an electrically pumped exciton‐polariton laser using InGaAs. Devices exhibiting CW lasing are especially promising candidates for electrical injection. More recently, topological defect states, specifically Su–Schrieffer–Heeger chains [245] shown in Figure 6c(ii), have been harnessed to achieve electrically pumped exciton‐polariton lasing, representing a major step toward practical coherent light sources. As illustrated in Figure 6d, the current state of research firmly establishes III–V semiconductors as viable platforms for electrically driven exciton‐polariton lasers, while opening an expansive research space for extending this capability to low‐dimensional semiconductors.

Generally, materials that exhibit continuous‐wave (CW) lasing can be considered potential candidates for electrically pumped polariton lasers. Most TMDs show lasing under CW excitation, as their high exciton binding energy and giant oscillator strength make them viable for lasing. However, defect density in TMDs plays a significant role in deteriorating exciton density. For designing electrically pumped exciton–polariton lasers, the electrical contacts should be transparent within the cavity; for instance, graphene contacts are transparent across the visible spectrum. If the contacts are placed outside the cavity, the cavity itself must be conductive. For example, GaAs/AlAs cavities are used in InGaAs‐based electrically pumped polariton lasers [258].

Most TMD polariton lasing has been observed at low temperatures, as Joule heating can potentially destroy the polaritons formed inside the cavity at room temperature. By carefully selecting the electron transport layer (ETL) and hole transport layer (HTL) to achieve balanced charge transport in the active material at high current densities, stimulated emission can be promoted over resistive heating.

Unlike TMDs, ion migration under an electric field is a major issue in perovskites. Field‐enhanced ion mobility is lower in 2D perovskites than in their 3D counterparts. In addition, synthesizing phase‐pure 2D halide perovskites is challenging. The inherent formation of different n‐phases leads to multiple radiative channels, thereby reducing spectral purity and coherence. Interestingly, the 2D perovskite FA_0.1_MA_0.9_PbBr_3_ has demonstrated polariton lasing at room temperature under CW excitation [229].

From a device fabrication perspective, planar waveguide designs are well suited for electrically pumped polariton lasers, as conventional p–i–n designs require complex DBR stacks and electrode architectures. Reducing the cavity volume decreases the number of supported modes, making compact cavity designs preferable for efficient lasing. Planar waveguides—such as distributed feedback (DFB) cavities or photonic crystal structures—can support bound states in the continuum (BICs) and are therefore ideal for achieving high quality factors. Such configurations can be electrically pumped using contact schemes similar to those employed in topological polariton lasers.

The previous section discusses exciton–polariton‐based lasers where strong coupling between the excitons and photons in a cavity leads to lasing upon exceeding the condensate population threshold. exciton‐polariton lasers facilitate low threshold lasing, which is a major requirement for applications demanding energy‐efficient integration of lasers, such as photonic integrated circuits and computing. Although there are no reports so far on electrically‐driven 2D exciton‐polariton lasers due to challenges such as imbalanced charge injection from electrical contacts, the Mott transition, and low carrier mobility, progress has been made in developing 2D exciton‐polariton‐based LEDs. These LEDs do not require condensation and show some interesting properties, such as polarized emission, which is one of the characteristics of lasing. Therefore, the device structures and emission properties of 2D LEDs will be discussed in the next section.

#### 3.1.3 2D Exciton–Polariton LED

**FIGURE 7:**
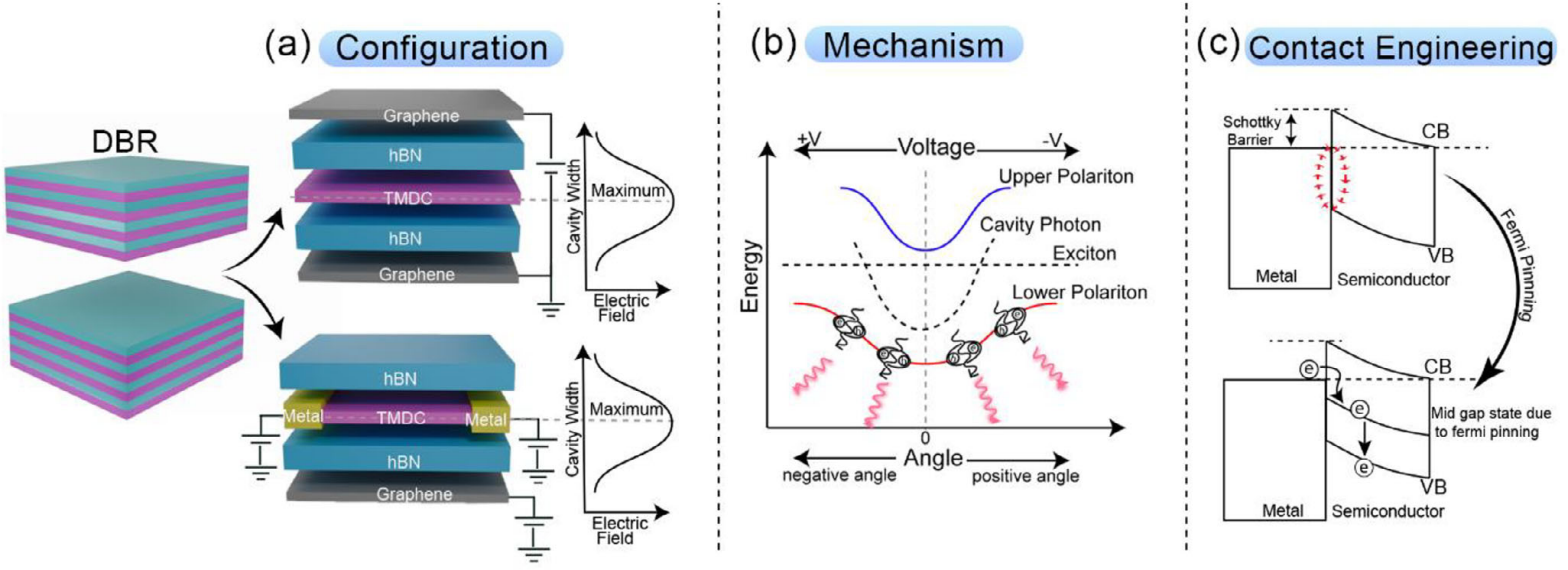
2D Exciton–polariton LED. (a) Diode configuration or transistor configuration of monolayers for obtaining EL from 2D materials, which are assembled inside a reflective mirror (DBR). The schematic of a graph showing the placement of 2D active material at the maximum point of the electric field inside the cavity. (b) E vs k for exciton‐polariton LED emission, where the angle of emission can be tuned by varying the voltage applied across the monolayer. (c) The interface between the monolayer semiconductor and the charge transport layers plays a crucial role in minimizing contact resistance. When a metal comes into contact with a semiconductor, a Schottky barrier typically forms due to differences in their work functions. Such an effect leads to Fermi‐level pinning, resulting in the formation of mid‐gap states at the interface. These states facilitate non‐radiative charge recombination, which can significantly impact charge transport and device performance.

As highlighted earlier, electrically driven exciton–polariton lasers in 2D materials have not yet been demonstrated. Nonetheless, important progress has been made toward this goal through the development of 2D exciton‐polariton LEDs, which reveal both promising findings and significant challenges. Figure 7a illustrates representative device configurations, where either a diode stack or a transistor stack is embedded within a DBR cavity. In one design, monolayer WS_2_ in a tunnel junction with hBN and graphene serving as electron and hole injection layers was sandwiched between a bottom DBR (SiO_2_/SiN_x_) and a silver top mirror [263]. This device exhibited exciton‐polariton EL with an EQE) of ∼0.1% [263], comparable to other electrically driven organic polariton LEDs [264]. The threshold voltage scaled with hBN thickness, reflecting the balance between tunneling current and the insulating nature of hBN. However, graphene‐based charge injection degraded the device quality factor due to passive absorption, reducing Rabi splitting. Thus, while hBN and graphene remain useful for charge injection, strategies to optimize light absorption near the exciton‐polariton emission or alternative injection layers are required.

Beyond conventional DBR‐based cavities, metasurfaces offer a promising pathway for realizing polariton LEDs, as they can support exciton–polariton modes without external mirrors while facilitating electrode integration. For instance, a metasurface cavity using MAPbI_3_ perovskites demonstrated electroluminescence under applied bias, with bound states in the continuum (BICs) enabling strong coupling and enhancing EL intensity by nearly 50‐fold [186, 265, 266]. Such results emphasize the importance of achieving high‐quality (Q) cavities capable of sustaining polaritons. The resulting exciton‐polariton electroluminescence exhibited angle‐dependent emission, tunable via source–drain voltage (Figure 7b). In these systems, injected carriers form excitons with limited in‐plane momentum, which relax to the lower polariton (LP) branch and emit through their photonic component. In addition to excitons, TMDs can host trions, an exciton bound with an additional electron or hole, that also undergo strong coupling. While trion–polariton emission has been reported in MoS_2_ through gate modulation in transistor‐like devices, trion–polariton LEDs remain unrealized and represent an unexplored opportunity [267].

The choice of contact material is also critical for efficient carrier injection into TMD monolayers. Poorly matched contacts can cause Fermi‐level pinning at the interface, leading to high resistance and reduced performance (Figure 7c). For example, inserting a thin bismuth layer before depositing silver electrodes reduces contact resistance by suppressing metal‐induced mid‐gap states [267]. Alternatively, van der Waals metals such as 1T′‐MoTe_2_, 1T′‐WTe_2_, 1T′‐PtSe_2,_ and 2H‐NbSe_2_ minimize Fermi‐level pinning through weak interfacial bonding with semiconductors [268]. The discovery of novel 2D metals that simultaneously alleviate Fermi pinning and provide light confinement as mirrors or cavities could be transformative for electrically driven polaritonic devices.

Efficient polariton LEDs require optical cavities with high reflectivity, low defect densities in the active material, and efficient charge injection from metal contacts into the semiconductor. Here, we compare the external quantum efficiency (EQE) of emerging polariton LEDs with that of conventional exciton‐based LEDs developed using 2D materials. This comparison helps identify the key bottlenecks in polariton LEDs. LEDs operating at high current densities strongly enhance many‐body interactions, giving rise to non‐radiative relaxation mechanisms such as exciton–exciton annihilation and Auger recombination, which substantially suppress radiative emission [269]. The EQE of these devices is inversely related to non‐radiative decay channels in the TMD layer. Over the past few years, various strategies have been employed to enhance the EQE of TMD‐based LEDs from ∼10^−^^5^ to approximately 1% [270]. Notably, oxygen plasma intercalation has recently been shown to enhance the EQE of MoS_2_ (0.02%) and WS_2_ (0.78%) by suppressing exciton–exciton annihilation [271]. Oxygen intercalation between TMD multilayers suppresses exciton–exciton annihilation. In addition, passivating the surface of WS_2_ using a non‐oxidizing organic superacid treatment has been shown to improve EL efficiency by up to 1.2% [272]. Beyond material quality, electrode contact engineering also plays a crucial role in determining the EQE. The choice of contact materials generally follows standard Ohmic contact criteria, wherein the metal work function should be lower than that of the semiconductor for n‐type contacts and higher for p‐type contacts. However, conventional metal deposition techniques, such as evaporation or lithography, often induce chemical reactions or defect formation in TMDs. Further, disordered metal–TMD interfaces introduce surface states that lead to Fermi‐level pinning and the formation of Schottky barriers, thereby hindering efficient carrier injection.

To address these challenges, researchers have explored 2D materials, particularly graphene, as vdW metal contacts. Graphene's dangling‐bond‐free surface, high in‐plane conductivity, and tunable work function via electrostatic gating make it an effective contact material for 2D semiconductors. Ultraclean interfaces can be achieved by dry transfer of graphene onto the active material. Despite these advantages, graphene suffers from relatively high series resistance when used as a contact, limiting its performance in practical devices. Consequently, while graphene is well‐suited for proof‐of‐concept demonstrations, alternative contact materials are required for scalable applications.

As promising alternatives, metallic TMDs—such as NbS_2_, NbSe_2_, VS_2_, VTe_2_, TaS_2_, PtSe_2_, PtTe_2_, NiTe_2_, CoS_2_, and ZrTe_2_—have been investigated as contacts [273]. Their higher conductivity compared to graphene, low growth temperatures, and composition‐tunable work functions make them attractive candidates. Both transfer‐based and vdW epitaxial growth methods have been employed to fabricate these contacts; however, the use of epitaxially grown metallic TMDs is constrained by the thermal budget of the active vdW materials in the device. To mitigate temperature‐induced defects, 2D/3D hybrid metallic stacks have been introduced for device contacts.

The dangling‐bond‐free and atomically thin nature of 2D materials enables the formation of clean, flat vdW interfaces with active TMD layers, suppressing Fermi‐level pinning while simultaneously acting as nearly transparent tunneling buffers for efficient carrier injection. Buffer layers such as insulating (hBN), semiconducting (TMD), and metallic (graphene) 2D layers have been widely employed to realize high‐quality vdW contacts between 3D metals and 2D TMD active materials. While the use of a 2D buffer layer allows modulation of the 3D metal work function, it also introduces additional tunneling and contact resistance. Therefore, direct contact between 3D metals and TMDs can be a viable strategy to minimize contact resistance. To prevent degradation caused by aggressive metallization, metals can be deposited onto a sacrificial layer (e.g., PDMS) and subsequently laminated onto the TMD. Considering the limitations of existing contact strategies, there remains a critical need for contact architectures that are stable, scalable, and capable of minimizing contact resistance [273]. The readers can refer to the review article specially focused on contact engineering for 2D materials, elsewhere [273]. Despite continuous improvements in the EQE of exciton‐based LEDs, the realization of ultralow‐power operation and pronounced angular dispersion within a single device architecture remains challenging.

Polariton‐based LEDs also suffer from similar limitations as discussed above for exciton‐based LEDs. For example, a WS_2_‐based polariton LED shows a relatively lower EQE of ∼0.1% with a turn‐on voltage of ∼4 V and a current density of 0.1 µA/µm^2^. In this device, graphene is used as the contact electrode. Owing to its zero bandgap and tunable Fermi level, graphene aligns with the conduction and valence bands of WS_2_, enabling both electrons and holes to be injected directly into the active layer. However, the presence of trapped air bubbles between the monolayers degrades interfacial contact, leading to increased contact resistance and consequently a higher turn‐on voltage [263]. One limitation of using 2D materials as charge injecting layer inside a cavity is the enhanced absorption in these layers at the resonant wavelength of the cavity [274]. As a result, reabsorption of the emitting photon by the passive layer further attenuates the intensity to the detector. Also, if the light extraction from the cavity is poor, the EQE will be further reduced. Better design strategies for making in‐plane cavities, such as lateral DBR coupled to waveguides, can be a promising design to outcouple the light for better EQE [275]. Another way to improve the polariton EQE is by designing the external cavity with 3D semiconductors with high electron/hole mobilities instead of dielectric cavities. The low carrier density inherent to polariton‐based LED operation limits radiative recombination, leading to lower EQE.

In the previous section, we discussed how exciton–polariton formation leads to LEDs exhibiting angle‐dependent emission intensity. Although the external quantum efficiency of exciton‐polariton LEDs (0.1%) is relatively lower than that of exciton‐based LEDs (1%), they exhibit enhanced color purity, which can be advantageous for high‐quality display technologies. Beyond these devices, the strong coupling in 2D materials can be explored for applications requiring sub‐band gap responses, such as photodetection, sensing, energy harvesting, and upconversion. In the next section, we will discuss exciton–polariton‐based photodetectors from single‐walled carbon nanotubes, perovskites, and other emerging semiconductors.

#### 3.1.4 2D Exciton–Polariton Photodetectors

**FIGURE 8:**
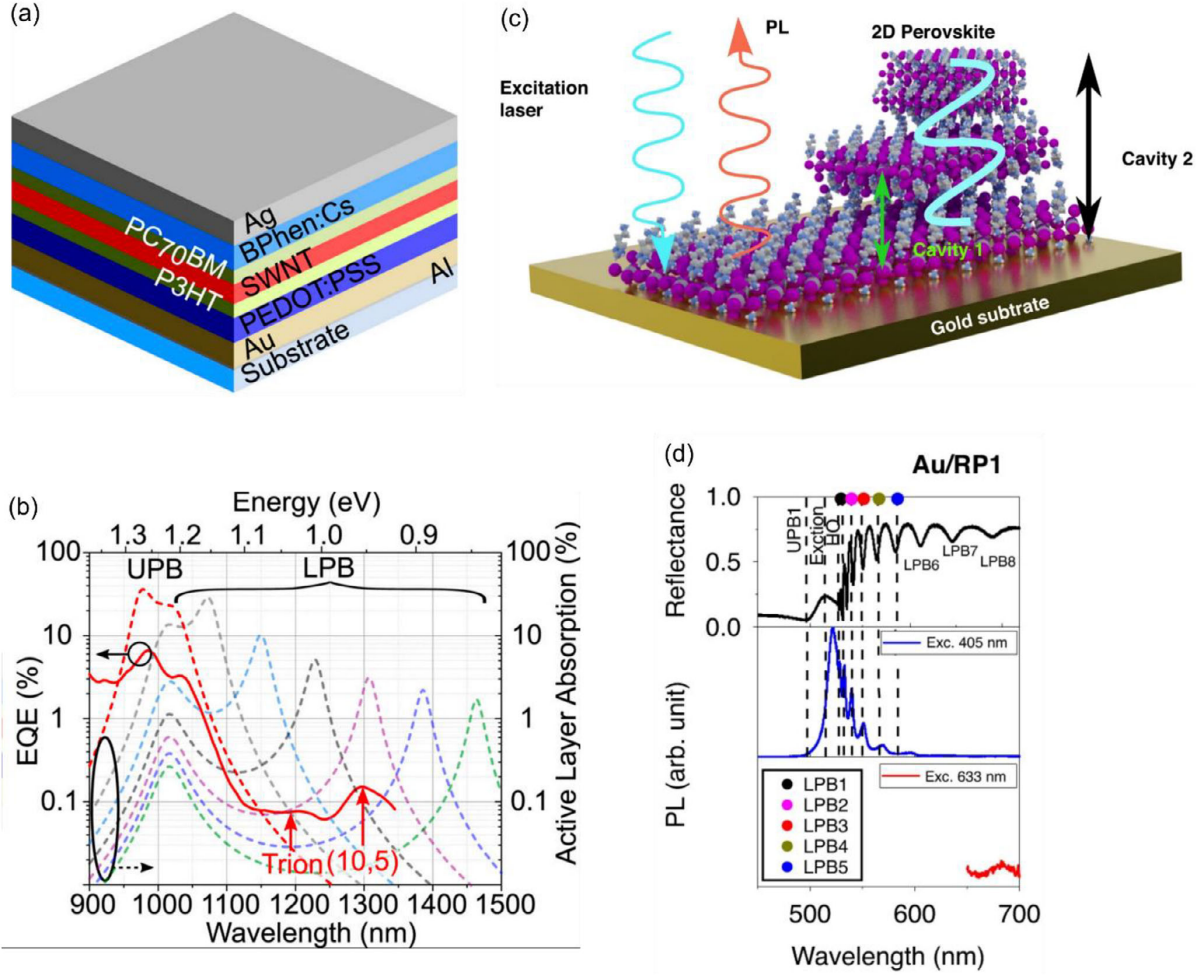
Exciton–polariton photodetectors (a) Device schematic for a photodiode utilizing P3HT: PC70 BM: SWNT as a flat heterojunction (FHJ) with Au–Ag cavity, PEDOT: PSS as HTL, and BPhen: Cs as ETL, (b) EQE (solid lines) of an FHJ photodiode and simulated absorption spectrum of a 10 nm thick SWNT layer (dashed lines). The red shift of the LPB corresponds to the increasing thickness of the P3HT: PCBM layer. The figures are reproduced with permission [276] 2020, AIP Publishing. (c) Schematic showing the exciton–polariton in 2D perovskites from the open cavity without DBR. (d) The sub‐bandgap states extending from the exciton peak at 520 to 700 nm are evident from the reflectance spectra. Reproduced with permission [274] 2024, Springer Nature.

Strong light–matter coupling arises when excitons interact with cavity photons, leading to hybridization and energy splitting that allow precise control over excitonic absorption and emission. This phenomenon has been demonstrated at room temperature in (6,5) single‐walled carbon nanotubes, where E11 excitons hybridized with near‐infrared cavity photons. Using two device architectures—bulk heterojunction (BHJ) and flat heterojunction (FHJ)—strong coupling was observed in Au–Ag cavities, yielding distinct upper and lower polariton branches (UPB and LPB) at 1.26 eV and 1.19 eV, respectively, with a Rabi splitting of 74 meV (Figure 8a). These polaritonic states enabled tunable photocurrent generation across the 1000–1300 nm spectral window (Figure 8b) [276]. Importantly, the LPB position can be modulated by varying cavity thickness, underscoring the need for external cavities in such systems.

In contrast, self‐hybridized exciton–polaritons have been reported in 2D Ruddlesden–Popper perovskites, achieved without the need for external cavities (Figure 8c) [274, 277]. Moreover, energy transfer from the UPB to multiple LPBs has been observed (Figure 8d), highlighting the richness of polaritonic dynamics in these systems. Optical engineering strategies can further enhance polariton absorption, opening pathways to advanced polaritonic photodetectors. TMDs also represent a promising platform for developing such polaritonic photodetectors [227].

From the above discussion, it can be noted that the exciton–polaritons‐based devices require an external cavity with a very high‐quality factor on the order of 10^2^ to 10^3^, depending on the oscillator strength of the 2D active material, to achieve strong coupling. The fabrication of external microcavities itself is very challenging, which necessitates alternative routes to achieve strong coupling using plasmons. By employing a plasmonic nanocavity, such as a nanoparticle on a mirror system, strong coupling between excitons and plasmons—known as plexcitons—can be achieved. However, the fabrication process is not highly deterministic. To enable more precise coupling between excitons and plasmons and to overcome the requirement of a cavity, surface plasmon polaritons can be utilized by simply placing the semiconductor on top of the metal surface. In this approach, the requirement of a cavity becomes optional, and strong coupling has been found suitable for various device applications such as photodetectors, chemical sensors, and single‐molecule sensors. In the following section, we will discuss the current state of the art of plexcitons‐based 2D materials for optoelectronic devices.

### 3.2 Plasmon–Polaritons‐Based Optoelectronic Devices

The free electron oscillation in metals, called a plasmon, when coupled with incident light, forms a plasmon–polariton. A surface plasmon polariton propagates between a metal and a dielectric material. These surface plasmon–polaritons enhance the electric field of the dielectric placed on top of the metal. While fabricating the device using plasmon–polaritons, we need to satisfy phase‐matching conditions owing to the difference between the in‐plane momentum of surface plasmons and free‐space photons. By tuning the angle of incidence of the photon and modulating the arrangement of dielectric material of high refractive index compensate for this difference and provide an efficient surface plasmon polariton excitation. If the surface plasmon polariton is formed at the interface of metal and DBR, then it is categorized as Tamm plasmon polaritons (TPPs). TPPs provide strong electric‐field confinement and sharp resonances without the need for complex patterning, offering a simpler and more scalable route for large‐area device fabrication [278] and have found immense application in the field of LEDs and photodetectors.

In layered 2D materials, strongly bound excitons remain stable at room temperature. Coupling these robust 2D excitons with the intense, confined fields of surface plasmons generates hybrid light–matter states, or “plexcitons” combining large oscillator strength, subwavelength confinement, sharp resonances, and strong nonlinearity. Experiments on monolayer and multilayer TMDs integrated with plasmonic nanocavities show clear anti‐crossing and Rabi splitting, including ∼80 meV in WSe_2_–gold systems and ultrastrong coupling with g/ω ≈ 0.16 in WS_2_ hotspot arrays [279]. These plexciton modes tightly confine optical fields, enhance excitonic emission and absorption, and exhibit extreme sensitivity to environmental changes, making them promising for devices [278].

#### 3.2.1 Plasmon–Polariton‐Based LEDs

Plasmon–polariton‐based LED can be classified into two categories according to coupling with plasmon (coherent oscillation of conducting free electron) and photon, surface plasmon–polariton (SPP) based LED, and TPP based LED. SPP enhances the internal quantum efficiency (IQE) of the device by the Purcell effect (weak coupling). Here we will discuss the SPP‐based LEDs. In LED device perspective, the use of an additional thin metallic layer or nanoparticle in the stack enhances the IQE of the device [280]. The excitation of SPP enhances the local electromagnetic field in the vicinity of the emitter material. This increased photonic density of states leads to an enhancement of the spontaneous emission rate via the Purcell effect. The enhancement of emission was observed in the organic fluorescent material when it was placed over a nano antenna [281]. A dye was placed over an aluminum nano structure gave enhancement as well as spatial coherence [282]. When selecting materials for plasmonic structures, noble metals are widely regarded as the most suitable choice because they exhibit relatively low optical losses and support well‐defined plasmonic resonances in the visible to near‐infrared spectral range [34].

In 2D materials, MoS_2_ was placed over an Ag disc and showed 12‐fold enhancement in photoluminescence [283]. Introducing 3 nm Al_2_O_3_ between the MoS_2_ and Ag nanoparticles leads to around 203‐fold enhancement in PL [284]. A plasmonic light‐emitting diode was fabricated using ITO and Al as the bottom and top electrodes, respectively. PEDOT: PSS and TPBi served as the ETL and HTL, while NPB (N, N′‐bis(1‐naphthalenyl)‐N, N′‐bis(phenylbenzidine)) was employed as a carrier‐blocking layer. CsPbBr_3_ nanocrystals were used as the emissive layer. Upon spin‐coating Ag nanoparticles onto the PEDOT: PSS layer, a 42% enhancement in electroluminescence intensity was observed compared with the reference device without plasmonic nanostructures [285]. Quasi‐2D perovskite LED also shows enhancement in EQE as Cu nanoparticles capped with polyvinylpyrrolidone [286] were used as plasmonic particles in the device. The dielectric medium placed between the emitter material and the metal nanoparticle plays a crucial role in modifying the local density of optical states, which in turn enhances the photoluminescence [287].

**FIGURE 9:**
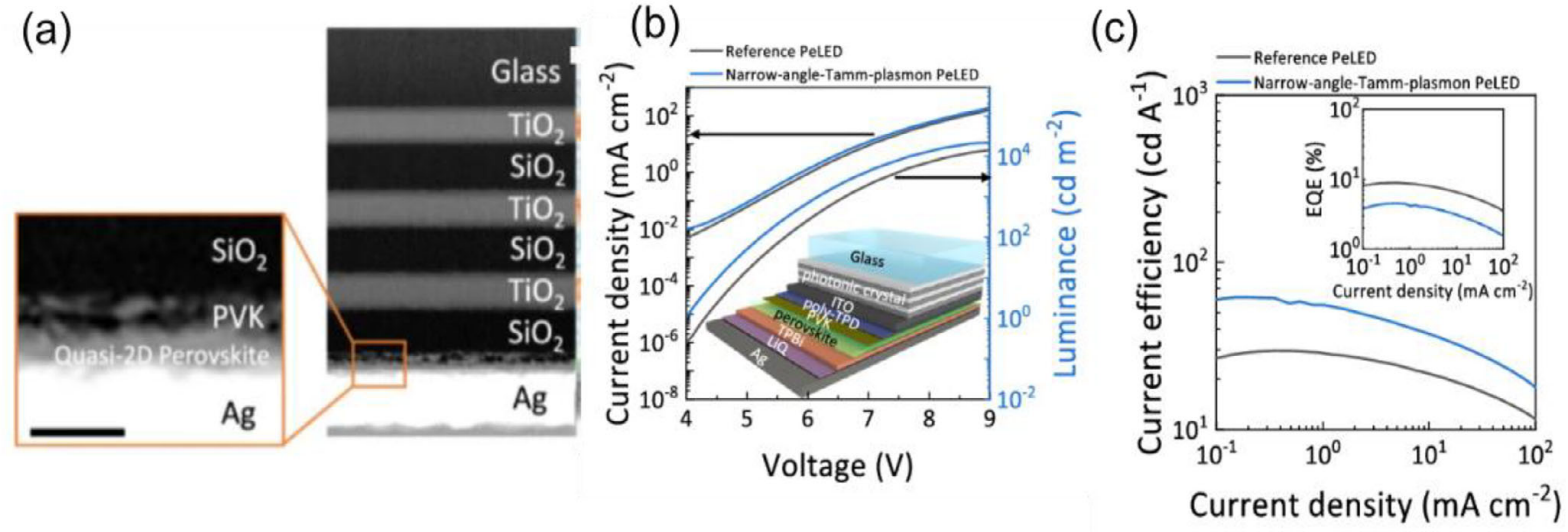
Plasmon‐polariton‐based LEDs. (a) Cross‐sectional high‐angle annular dark field scanning transmission electron microscopy image of the perovskite‐based Tamm plasmon structure, (b) Current density/luminance vs voltage of the reference PeLED and narrow‐angle Tamm‐plasmon‐driven PeLED, and (c) Current efficiency vs. current density with the inset depicting the EQE vs. current density considering emissions over all angles (all figure panels are reproduced under terms of the CC‐BY license [288] 2024, Ooi et al., Springer Nature).

Integrating TPPs into perovskite light‐emitting diodes (PeLEDs) has been shown to significantly improve device performance. TPPs form at the interface between a metal layer (e.g., silver) and a photonic crystal, producing strong light confinement (Figure 9a). Their interaction with quasi‐2D perovskite layers sharpens the angular emission profile, reducing the full‐width at half‐maximum (FWHM) from 143.9° to 36.6° and enabling more directional emission, which is advantageous for display and optical communication applications. Moreover, TPPs enhance forward‐directed light output by 80%, thereby increasing brightness and current efficiency without additional energy input (Figure 9b). The EL linewidth also narrows from 22.4 nm to 12.1 nm, improving color precision, an essential feature for high‐purity display technologies.

The device architecture comprises a TiO_2_/SiO_2_ photonic crystal, a quasi‐2D perovskite layer, and a silver layer that together form the DBR, enabling strong light–matter coupling [288]. A thin silver layer beneath the DBR supports TPP formation. Compared to a reference PeLED lacking photonic crystal layers, the TPP‐enhanced device demonstrates markedly improved performance, achieving higher forward luminance (21 800 cd/m^2^ vs. 14 500 cd/m^2^) and current efficiency (61.8 cd/A vs. 29.6 cd/A), attributed to enhanced directional emission (Figure 9c). However, the EQE shows a slight reduction due to optical losses associated with plasmon coupling. Overall, integrating TPPs into PeLEDs provides a promising route for realizing narrow‐linewidth emissions, offering a pathway toward electrically driven plasmonic lasers.

In the previous section, we discussed plasmon–polariton‐based LEDs. The discussion demonstrated directional luminescence enhancement in polariton LEDs via plasmonic field concentration, as evidenced by exciton–plasmon hybridization. By optical reciprocity, strong coupling in plasmon–polaritons equivalently enhances photodetection through amplified light–matter interaction and extends spectral absorption by creating sub‐bandgap states. These quasiparticles are highly sensitive to their surrounding dielectric environment. This tunable, material‐responsive characteristic establishes plasmon–polaritons as strong candidates for integrated photodetector architectures and next‐generation sensing systems. In the next section, we will examine photodetectors based on plasmon–polaritons.

#### 3.2.2 Plasmon–Polariton‐Based Photodetectors

**FIGURE 10:**
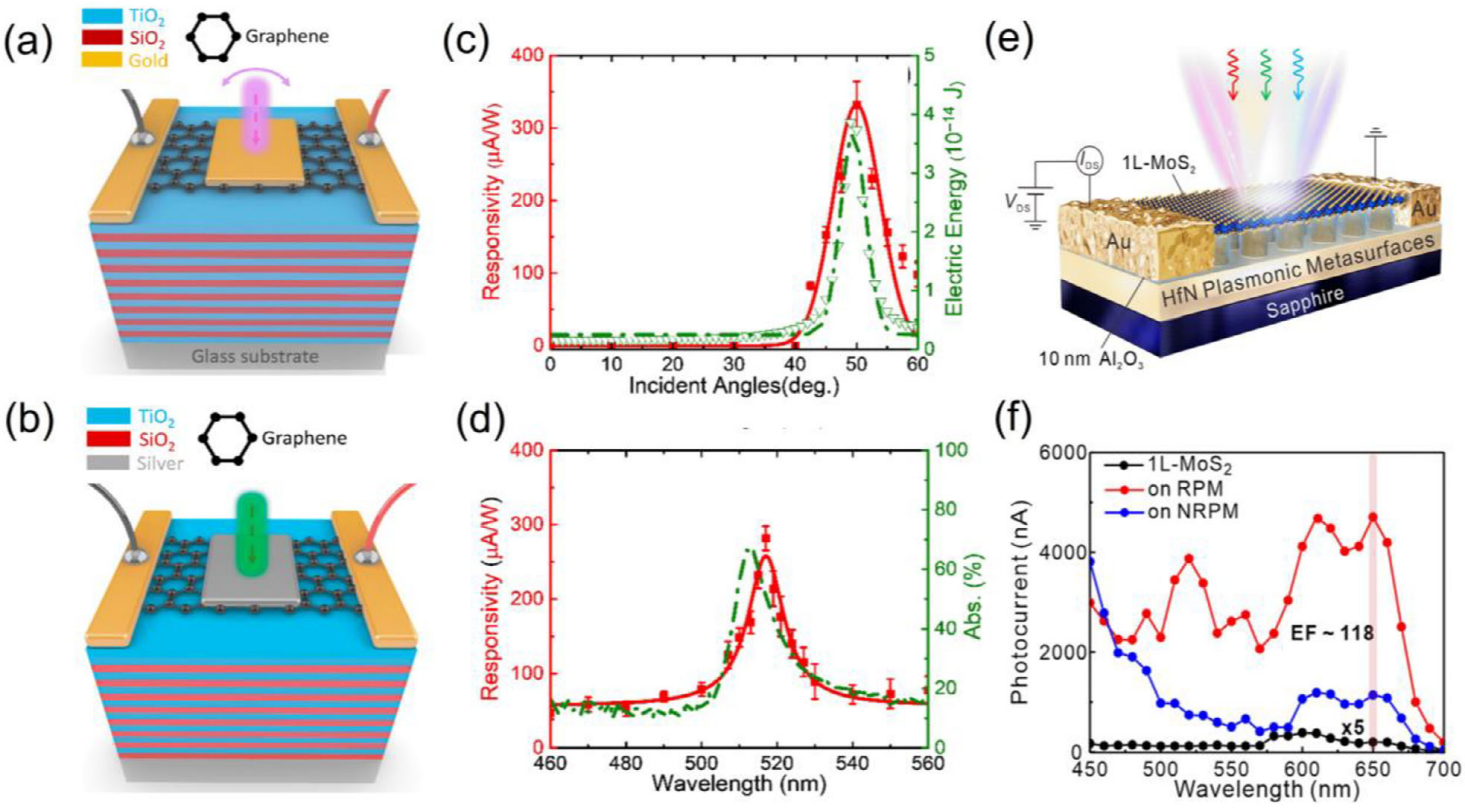
2D plasmon polariton photodetectors. (a,b) Schematic of graphene Tamm plasmon polariton photodetector with Au and Ag (Reproduced under terms of the CC‐BY license. [289] 2023, Huang et al., MDPI) (c) Angle‐selective and (d) wavelength‐selective responsivity from the device operating in the visible region (Reproduced under terms of the CC‐BY license. [289] 2023, Huang et al., MDPI). The red and green line corresponds to responsivity and simulated electrical energy, respectively. (e) Schematic of SPP TMD photodetector (Reproduced with permission. [292] 2024, American Chemical Society) (f) Photocurrent enhancement using SPP for monolayer TMD photodetector (Reproduced with permission. [292] 2024, American Chemical Society).

Devices exploiting plasmonic states—SPPs and TPPs—have been widely applied in nanoantennas, metasurfaces, and biosensors. For instance, graphene–TPP devices (Figure 10a,b) have enabled angle‐ and wavelength‐selective miniaturized LiDAR systems operating in the near‐infrared (NIR) and visible regions, achieving enhanced photocurrent responsivity compared to control devices without TPPs (Figure 10c,d) [289]. Graphene photodetectors coupled with Ag nanowires have also been shown to detect long‐range SPPs with high responsivity (∼17 mA/W), making them attractive for nanophotonic circuits [290]. Similarly, NIR photodetectors based on Au nanoparticle–decorated graphene on silicon nanowires demonstrated improved light trapping, high responsivity (1.5 A/W), detectivity (2.54 × 10^14^ Jones), and fast response times. Responsivity has been further enhanced in graphene SPP detectors integrated with nanograting and C‐shaped plasmonic arrays, reaching ∼15 mA/W.

Beyond graphene, TMD monolayers integrated with plasmonic metal structures have supported plasmon–polariton states that can be tuned between strong and weak coupling regimes by applying an external gate voltage [291]. Polariton photodetectors fabricated using resonant plasmonic nitride‐based structures exhibited photocurrent enhancements up to 100‐fold compared to pristine MoS_2_ thin films (Figure 10e,f) [292]. Comparable improvements have also been observed in plasmonic Ge/Si quantum dot detectors integrated with gold film hole arrays, achieving ∼30‐fold enhancement in mid‐infrared responsivity at 5.4 µm [293].

Plasmon‐enhanced detection has also proven valuable across other spectral ranges. In the NIR, organic photodiodes incorporating charge‐transfer states in dielectric microcavities with a 25 nm silver layer achieved narrowband responses and 17% external quantum efficiency at 880 nm [294]. In the UV, ZnO [295] and β‐Ga_2_O_3_ [296]‐based MSM devices decorated with Ag or Au nanoparticles exhibited 100‐fold increases in responsivity along with faster response times. Furthermore, photoelectrochemical UV detectors employing Au nanoparticle electrolytes demonstrated significant improvements in responsivity and sensitivity, attributed to plasmon‐enhanced light harvesting [297]. Collectively, these advances underscore the versatility of plasmonic devices and highlight their potential for a wide range of optoelectronic applications spanning NIR, mid‐IR, and UV detection.

Here, in Table 4, we can see the different parameters for conventional and plasmon–polaritons‐based photodetectors.

**TABLE 4:** Comparison of performance for conventional and plasmon–polariton 2D photodetectors.

Device Type	Device stack	Responsivity (A/W)	Wavelength (nm)	Refs.
**Conventional 2D material‐based photodetector**	Gr/(BA)_2_PbBr_4_‐graphene	2.1 × 10^3^	512	[298]
	Au/(PEA)_2_PbBr_4_/Au	5.4 × 10^−3^	550	[299]
	Bi‐layer graphene	6.1 × 10^−3^	1550	[300]
	IL‐MoS_2_	8.8 × 10^2^	561	[301]
	MoS_2_	5 × 10^−2^	532	[302]
**Plasmon**–**polariton‐based photodetector**	Photonic crystal/Ge/GeO_2_/TiO_2_	4.2 ×10^−2^	905	[303]
	TiO_2_/SiO_2_/Au/Graphene/Au	2.71 × 10^−4^	517	[289]
	MoS_2_/Ag nanowire	59.6	532	[302]
	TiO_2_/SiO2/Au/Graphene/Au^a^	3.3 × 10^−7^	945	[289]
	Au NP/Graphene	1.6 × 10^−4^	1300–1800	[304]

^a^ —Angular selectivity = 60°

Although a plasmonic cavity provides strong coupling to the excitons, it has some challenges as well. Plasmonic resonators suffer from radiative damping and ohmic losses in metals, yielding Q‐factors significantly lower than dielectric cavities. The ohmic loss is a major challenge for photodetector also, as the absorption increases; however, the energy is converted into heat rather than photocurrent. Also, strong exciton‐SPP coupling requires substantial wavefunction overlap between excitons and spatially confined SPP fields, which is challenging given the short‐ranged nature of SPP electric fields and the difficulty in achieving efficient coupling with few or multiple excitons.

Until now, the devices we discussed were based on quasiparticles and phenomena that are governed by either optical or electrical excitations. Besides electrical excitation, one can also use magnetic excitations to design room‐temperature superconductors, magnetic spins for quantum technologies, and spintronic‐based devices. Next, we will discuss the spin‐based phenomenon and quasiparticle called magnon–polariton whose properties can be modulated using magnetic fields. Magnon–polariton‐based device facilitates external magnetic field as an extra degree of freedom to tune the device, which can find applications in terahertz wave generation and other spintronic applications.

### 3.3 Magnon Polaritons

Moving beyond exciton and plasmon‐based polaritonic systems, cavity spintronics has emerged as a promising frontier for light–matter hybridization. Within this framework, magnon–polaritons, a hybrid quasiparticle formed through the strong coupling between magnons, the collective excitations of magnetic order, and photons, have gathered increasing interest.

Cavity spintronics explores strong coupling between spin ensembles (magnons) and photons confined in a cavity. This makes cavity spintronics an interface between spintronics and quantum phenomena. The criteria for strong magnon–polaritons are similar to exciton–polaritons as discussed above, where the interaction strength (*g*) exceeds the magnon (γ) and cavity (κ) dissipation rates, producing clear anti‐crossing in the microwave transmission spectrum. The interaction between magnon and photon can be represented by cooperativity, *C* = 4*g* ^2^/κγ ≫ 1 serving as a figure of merit for coherent operation. Materials supporting magnon–polaritons can be ferrimagnetic insulators (Yttrium Iron Garnet (YIG)), ferromagnets (Ni_80_Fe_20_, metals such as Co, Fe), multiferroics (BiFeO_3_), antiferromagnetic materials (NiO, FeF_2,_ 2D materials such as CrSBr), and very recently, 2D magnetic materials such as CrI_3_, Fe_3_GeTe_2_, and Cr_2_Ge_2_Te_6_.

**FIGURE 11:**
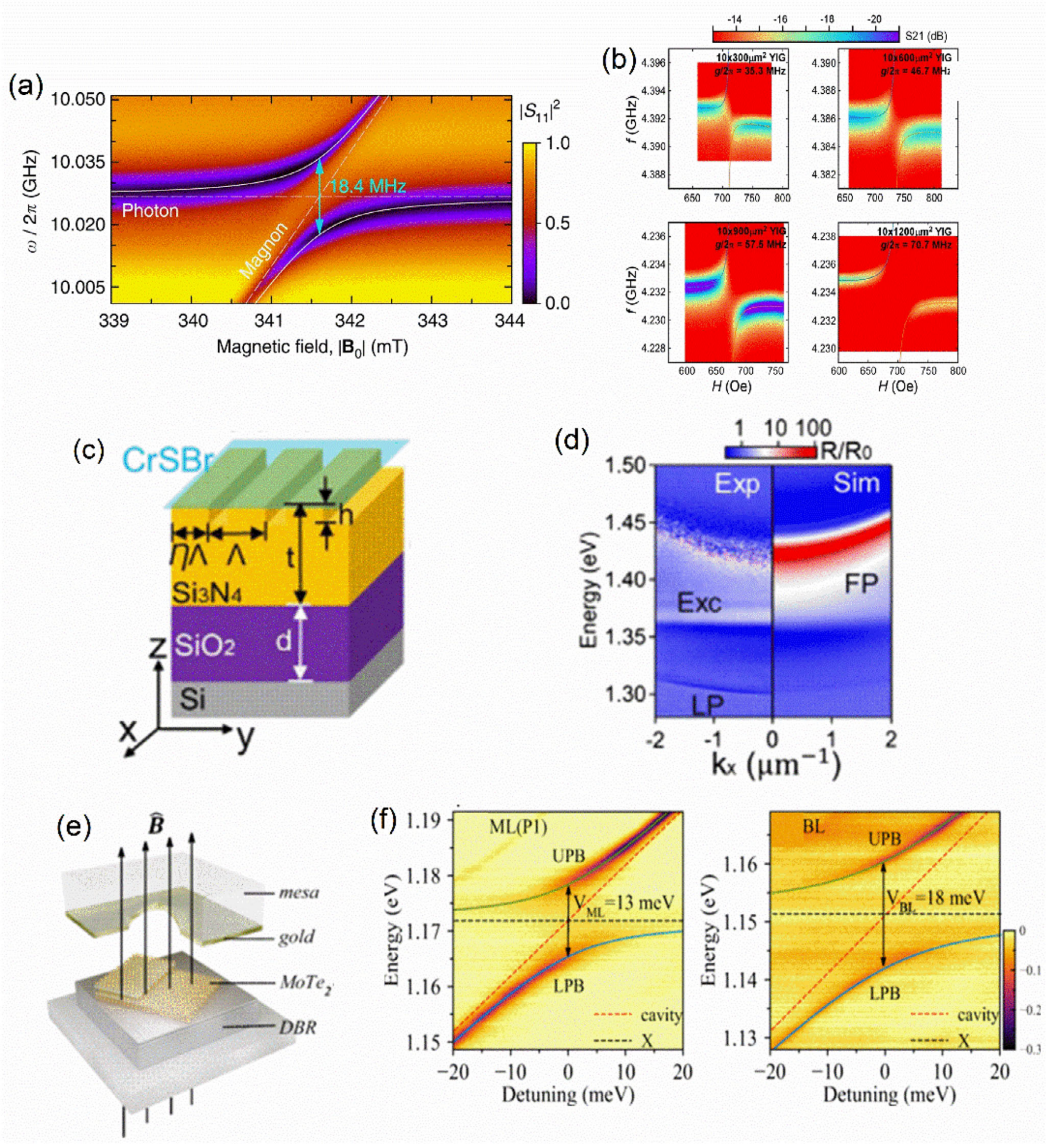
Magnon polaritons (a) Maximum coupling of the TE102 mode to the magnon occurs at a YIG sphere displacement of 11 mm, with a fitted coupling strength of 9.2 MHz (Reproduced with permission [307] 2017, Springer Nature) (b) Microwave transmission in Nb resonators coupled to YIG strips of different dimensions shown as a function of microwave frequency and in‐plane magnetic field at 2 K (Reproduced with permission [309] 2023, American Chemical Society) (c) Schematics for a seven‐layer CrSBr stack on Si_3_N_4_ to study the anisotropic behavior of exciton–magnon–polariton system (Reproduced with permission [325]2025, American Physical Society). (d) The observation of the lower polariton branch shows the strong coupling nature in the CrSBr on the Si_3_N_4_ system. (Reproduced with permission [325] 2025, American Physical Society). (e) The schematics for the mono and bilayer TMD in an open cavity in a magnetic field. (Reproduced with permission [335] 2025, American Physical Society). (f) Differential reflectivity as a function of cavity detuning with monolayer (P1) and bilayer, with exciton energy of 1.1719 eV and 1.1513 eV, and Rabi splitting of 13meV and 18meV, respectively (Reproduced with permission [335] 2025, American Physical Society).

Studies have shown that magnon dynamics can be engineered through material choice and device architecture to enable nonvolatile, low‐power signal processing beyond charge‐based electronics. In hybrid quantum and photonic schemes, theoretical work predicts that antiferromagnetic insulators with low Gilbert damping can host optical and microwave magnon modes that coherently couple to cavities, enabling frequency conversion with efficiencies limited primarily by magnon linewidth and cavity loss rather than electronic dissipation [305, 306]. Macroscopic YIG has served as a conventional 3D system to study magnon–polaritons. A coupling strength of 9.2 MHz and a magnon linewidth of 1.5 MHz has been achieved by enabling magnetic‐field‐tunable hybrid eigenstates at room temperature (Figure 11a) [307]. Extending beyond two‐mode hybridization, triple strong coupling among ferromagnetic magnons, microwave photons, and long‐lived phonons was demonstrated in YIG cavity systems. By exploiting coherent perfect absorption to suppress polariton decay, a polaromechanical cooperativity ∼9.4 × 10^3^ was achieved, enabling efficient three‐way energy exchange and revealing cavity magnon–polariton as versatile mediators for multimode coherent control [308].

Placing these YIGs on superconductors can further enhance the magnon interaction with the microwave photon. By utilizing a high‐quality YIG thin films on a diamagnetic Y_3_Sc_2_Ga_3_O_12_ substrate integrated with superconductor resonators led to strong microwave photon–magnon coupling at a few Kelvin temperatures (Figure 11b). This highlights a decisive benefit over metallic ferromagnets by strongly suppressing magnon decay, making them especially well‐suited for on‐chip hybrid quantum platforms that integrate magnonic waveguides with superconducting microwave resonators and qubits for quantum information processing at millikelvin temperatures [309]. Building on cavity‐based quantum systems, YIG has been used to realize functional devices. A nonvolatile three‐terminal lateral magnon field‐effect transistor was realized by integrating a YIG on a ferroelectric substrate with patterned injector, gate, and detector electrodes, achieving nonvolatile gating of magnon transmission with a high on/off ratio of ∼400% at room temperature [310].

The observation of nonlinear magnonic phenomena in YIG further expands its functional scope. Millimetre‐scale YIG sphere embedded in microwave cavities has shown that strong magnon–photon coupling with a coupling strength of 41 MHz, combined with intrinsic magnon Kerr nonlinearity, can induce hysteretic switching between distinct polariton states. By tuning the orientation of the YIG crystal, bistable behaviour emerges, enabling stable information retention [311]. Advancing further, a multistability and memory functionality has been observed in a three‐mode cavity magnonic YIG system, which allows long‐time memory of 5.11 s and ternary logic operations using the history‐dependent occupation of each branch, creating a new path toward cavity magnonics‐based memory and computing [312]. These findings open new avenues for developing energy‐efficient magnon‐based logic devices and highlight their potential for unconventional computing paradigms, including neuromorphic architectures. They also highlight key challenges, such as magnon attenuation, limited amplification mechanisms, and the difficulty of integrating multiple magnonic elements into scalable, CMOS‐compatible circuits [313].

Despite the wide range of functionalities in YIG systems, from ultrafast information processing, non‐reciprocity, and microwave‐to‐optical transduction [314, 315, 316, 317, 318, 319, 320], their reliance on bulk magnetic crystals and millimeter‐scale cavities limits the miniaturization and integration with modern electronics and photonics. To address these limitations, two‐dimensional van der Waals (vdW) magnets, with their atomic‐scale thickness and natural compatibility with on‐chip integration, have emerged as a promising material of interest. Coupling these atomically thin systems to microwave resonators with small mode volumes and strong magnetic‐field confinement enables access to magnon–polariton regimes, extending functionalities beyond those achievable with bulk YIG platforms [321, 322, 323].

Two‐dimensional van der Waals antiferromagnetic semiconductors, particularly CrSBr, have emerged as a versatile platform for exploring magnon–polariton physics. Coherent coupling between magnons and excitons in CrSBr has been demonstrated, where optically launched magnons modulate exciton energies and propagate over micrometer scales with nanosecond coherence, establishing an optical window into spin dynamics in 2D magnets and suggesting routes toward optically accessible magnonics and spintronic interconnects. Strong coupling has been realized between antiferromagnetic magnons in CrSBr and microwave photons confined within a niobium on‐chip resonator, achieving a coupling strength of 0.27 GHz and a cooperativity of 4.78. This enables transfer of information between spin and electromagnetic degrees of freedom, offering a tunable platform beyond traditional ferrimagnetic materials. Such coupling holds promise for achieving efficient single‐photon microwave‐to‐optical photon transduction [324]. A 7‐layer CrSBr integrated on an anisotropic silicon nitride photonic crystal cavity (Figure 11c) demonstrates exciton–polaritons with strongly coupled atomic, spin, and photonic anisotropies, exhibiting vacuum Rabi splitting of 116 ± 3 meV and coupling strength of 58 ± 2 meV [325]. This strong coupling is evidenced by the visibility of the lower polaritonic branch in Figure 11d. Remarkably, polariton polarization rotates 18 to 42° from cavity/exciton modes and is tunable by tens of degrees via cavity Q‐factor, detuning, magnetic field, and temperature. The high saturation density and small Bohr radius (1.3–4.8 Å) confirm quasi‐1D exciton character, while rapid mode disappearance above Néel temperature reveals a strong correlation between excitons and magnetic order. These results enable compact on‐chip polarized light sources, tunable photonic devices, and exploration of spin–charge–light interactions in 2D magnets. Beyond cavity polaritons, CrSBr also supports hyperbolic exciton polaritons with subdiffractional confinement that are enhanced by anisotropic excitonic responses and magneto–electronic effects, pointing toward nanoscale manipulation of light and excitons [326]. Magnons in CrSBr can also mediate nonlinear exciton–exciton interactions by adjusting spin canting to induce nonlinear optical responses. This introduces a mechanism for magnetically tunable optical nonlinearity and suggests that magnons can function as active intermediates bridging optical and microwave domains [327].

The magnon–polariton physics is not limited to antiferromagnets. Ferromagnetic vdW materials such as Cr_2_Ge_2_Te_6_ have also been coupled to superconducting microwave resonators, demonstrating high‐cooperativity photon–magnon hybridization and confirming that atomically thin magnets (∼11 nm) can sustain coherent magnon–photon polaritons suitable for on‐chip integration [328]. Magnon–polaritons were also realized by coupling graphene plasmons with magnons in 2D ferromagnetic CrI_3_, achieving a large Rabi splitting (∼100 GHz) and enabling ferromagnetic resonance measurements via attenuated total reflection [329]. Together, these findings establish that 2D magnetic semiconductors can host hybrid polaritonic states with strong coupling, tunability via magnetic field, and interactions among spin, charge, and light degrees of freedom, providing a material framework for devices such as low‐power magnonic logic elements, tunable photonic components, and on‐chip coherent transducers.

Parallel to developments in low‐dimensional systems, surface magnon–polaritons have been explored in bulk magnets, particularly in the terahertz (THz) regime. Experimental evidence of THz wave propagation mediated by surface magnon–polaritons has been reported in anisotropic antiferromagnets near spin resonances [330]. Nonreciprocal surface magnon–polaritons have also been exploited in twist‐engineered ferromagnetic insulator interfaces to achieve tunable near‐field heat transfer, enabling mechanically reconfigurable control over spin–photon interactions with applications in thermal management and nonreciprocal energy transport [331]. Theoretically, thin films of negative‐permeability materials such as MnF_2_ and FeF_2_ are predicted to support surface magnon–polaritons with extreme wavelength compression exceeding four orders of magnitude and dramatic enhancement of THz spin–flip transitions, offering prospects for ultrafast THz spin control [332]. The realization of THz magnon–polariton physics has also been demonstrated in TmFeO_3_, where magnon–polaritons dominate THz wave propagation near quasi‐antiferromagnetic resonances, forming distinct upper and lower polariton branches observable via time‐domain THz spectroscopy [333]. More recently, strong photon–magnon coupling above 1 THz has been achieved at room temperature in NiO using Fabry–Pérot cavities, opening new directions for THz spintronics and high‐frequency quantum information technologies [334].

In addition to the above systems, TMDs provide a complementary materials platform owing to their strong light–matter coupling and intrinsic spin–valley locking, making them well‐suited for cavity quantum electrodynamics. TMD polaritons enable exploration of bosonic condensation, correlated magnetism, and magneto–optical effects. For instance, studies of MoTe_2_ monolayers and bilayers reveal enhanced polaritonic Zeeman splitting and oscillator strength, with bilayers exhibiting a 38% increase in Rabi splitting and improved polariton relaxation dynamics [335]. The device schematics are shown in Figure 11e, while the Rabi splitting is visible in Figure 11f. Taken together, these advances indicate that realizing scalable magnon–polariton devices will depend critically on heterostructure engineering to maximize mode overlap, integration with on‐chip microwave and optical resonators, and materials optimization to suppress damping and extend coherence toward room‐temperature operation.

These studies establish magnon–polaritons as a unifying framework from microwave, optical, and terahertz regimes across diverse material platforms. The transition from bulk ferrimagnetic systems to atomically thin magnetic layers shows a clear pathway toward scalable, low‐loss, and highly tunable architectures. Continued progress in materials synthesis, interface engineering, and cavity design is expected to enable next‐generation quantum technologies, reconfigurable photonic systems, and energy‐efficient spin‐based information processing.

In previous sections, we have discussed various types of quasiparticles such as excitons, exciton–polaritons, plexcitons, and magnon–polaritons. We also discussed various devices using these quasiparticles, such as solar cells, photodetectors, LEDs, and lasers. However, the experiments that require probing these quasiparticles also play an important role in understanding the properties and limitations of the device architecture. For example, electron energy loss spectroscopy (EELS), which measures the momentum and energy of the charge carriers, such as electron oscillations in plasmons, provides important insight about the Rabi splitting and bright/dark plasmonic modes [336]. Now we will turn our focus to these experimental methods that we need to probe these quasiparticles and their properties.

## 4 Electron Microscopy and Spectroscopy in Analyzing Electromagnetic Modes of Optical Materials

From the above discussion, it is evident that strong coupling plays a crucial role in advancing device performance. To design next‐generation high‐performing devices, it is essential to image and detect these coupled states at the nano‐ and atomic scales. Recent advancements in electron microscopy and spectroscopy have greatly expanded our ability to probe electromagnetic modes in optical materials. Techniques such as electron energy loss spectroscopy (EELS), cathodoluminescence (CL), and ultrahigh‐energy‐resolution EELS are particularly powerful for studying light–matter interactions at the nanoscale. These methods enable direct visualization and quantitative analysis of plasmonic resonances, phonon interactions, and strong coupling phenomena with unprecedented spatial, temporal, and energy resolutions [337, 338].

### 4.1 EELS, CL, and Ultrahigh‐Energy Resolution Spectroscopy

**FIGURE 12:**
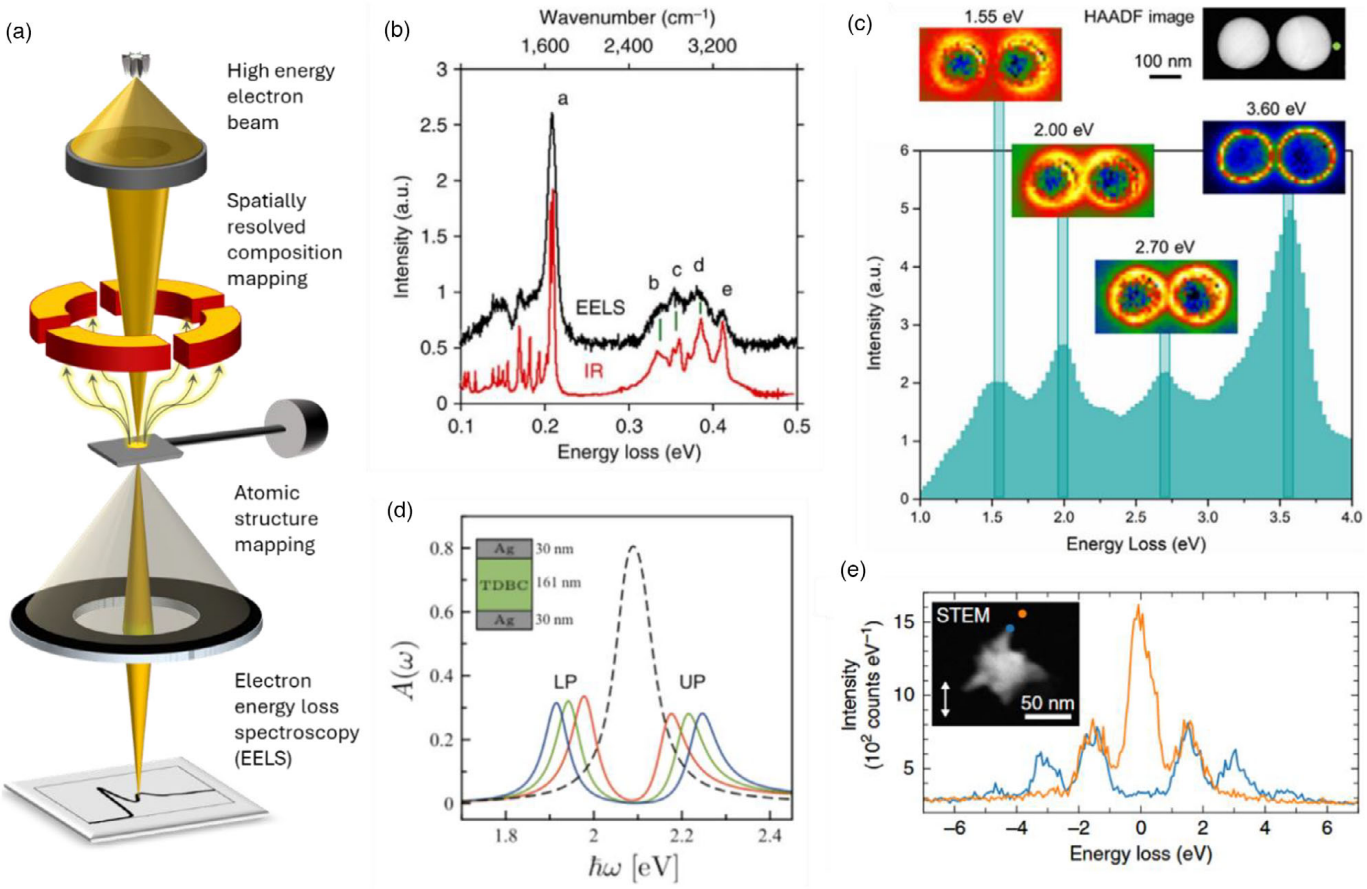
Correlative Optical and Electron Microscopy. (a) Schematic demonstrating the working principle of EELS, (b) Correlation between IR spectroscopy and EELS in mapping atomic vibrations. Reproduced with permission [339] 2016, Springer Nature (c) Spatial mapping of low‐energy optical modes between 100 nm diameter nanoparticles separated by 20 nm. Reproduced with permission [340] 2018, American Chemical Society (d) Rabi splitting of optical modes into lower and upper polariton states between two silver mirrors. Reproduced with permission [341] 2020, AIP Publishing (e) Mapping of the electron beam stimulated electron–photon interactions in a gold nanostar using EELS Reproduced with permission [342] 2021, Springer Nature.

EELS and CL serve as cornerstone techniques for nanoscale optical characterization. In EELS, the energy lost by incident electrons during interactions with a sample provides direct information on local optical responses, including plasmon resonances and phonon modes (Figure 12a). In contrast, CL detects the light emitted upon electron excitation, offering insights into radiative optical processes and emission pathways [337, 338].

The strength of EELS and CL lies in their ability to probe spatially localized phenomena with sub‐nanometer precision. Recent advances, particularly in hyperspectral imaging, have enabled mapping of optical properties with unprecedented detail. For example, EELS can reconstruct the local density of photonic states (LDOS), a key parameter for understanding electromagnetic field distributions [337]. Rez et al. demonstrated that molecular vibrations in biological samples can be mapped using EELS, with results similar to infrared spectroscopy. Achieving meV‐scale energy resolution in EELS, combined with the spatial precision of electron microscopy, makes it possible to reveal vibrational and optical features at nanometer length scales that are otherwise inaccessible with conventional optical microscopy (Figure 12b) [339].

Parallel progress in monochromator technology has further expanded the capabilities of EELS, pushing energy resolution into new regimes. These advancements enable direct exploration of low‐energy excitations such as vibrational modes and strongly coupled hybrid states. High‐resolution EELS has been instrumental in studying phenomena including plasmon–exciton interactions and phonon dispersion mapping at the nanoscale. For instance, high‐energy‐resolution STEM–EELS reveals how defects and interfaces affect electromagnetic behavior, while in doped semiconductors, it correlates local doping concentrations with plasmonic responses, advancing our understanding of structure–function relationships in optical materials [338].

### 4.2 Plasmonic Resonances, Strong Coupling, and Hybrid Modes

Electron microscopy has made remarkable progress in probing plasmonic resonances, which are central to understanding light–matter interactions in metallic nanostructures. These resonances, arising from collective electron oscillations, can be tuned by modifying the geometry, size, or composition of the material [343, 344]. Using EELS, dipole–quadrupole interactions in nanoparticle arrays have been extensively studied (Figure 12c), revealing the evolution from individual particle resonances to collective modes—findings that are pivotal for the design of metamaterials and photonic devices [344]. Toward this end, EELS has been applied to probe excitons by Suenaga's group [345, 346] back in 2015 on MoS_2_ and MoSe_2 _heterostructures. They observed exciton peaks with 10 nm spatial resolution and attributed the splitting of the peaks to spin–orbit coupling. Furthermore, momentum‐resolved (q‐EELS) has been used to map exciton polaritonic modes in thin films of WSe_2_ [347]. More recently, Susarla et al. [348] demonstrated that intralayer excitons can be mapped with sub‐nanometer resolution within complex Moiré patterned unit cells of WS_2_‐WSe_2_.

Beyond plasmonics, strong coupling phenomena—where light and matter coherently exchange energy—have drawn significant attention in quantum optics and photonics. EELS and CL have enabled direct visualization of vacuum Rabi splitting and the formation of polaritonic states in nanostructures (Figure 12d,e) [342, 343]. Advanced EELS techniques have also uncovered hybrid modes such as plasmon–phonon polaritons, illustrating the interplay between electronic and vibrational excitations. These insights are not only fundamental for understanding nanoscale interactions but also critical for developing future applications in energy harvesting and optoelectronic devices [338, 344].

## 5 Perspectives and Future Directions

We have reviewed recent progress in excitonic and strong light–matter coupling‐based optoelectronic devices, and summarize our perspective while proposing future directions.

**Solar Cells**: For TMD‐based solar cells, wafer‐scale growth of monolayer TMDs has been demonstrated; however, the reported power conversion efficiency (PCE) for single‐junction cells—whether monolayer, multilayer, or mixed‐dimension—remains around ∼10%. Further improvements require high‐quality, defect‐free TMDs and advanced electrode engineering to minimize Schottky barriers. Progress is being made in developing optimized p‐ and n‐type contacts. Soft deposition techniques are also needed to reduce Fermi‐level pinning caused by direct metal deposition. Promising results include electron‐beam deposition of Pd and Pt for p‐type contacts on multilayer TMDs, achieving near‐ideal van der Waals interfaces without chemical reaction. Printing techniques such as aerosol‐jet printing may provide scalable alternatives. For 2D perovskites, mixed 2D/3D systems outperform pure 2D counterparts in PCE, though the larger bandgaps of 2D perovskites limit absorption. Nevertheless, their sharp excitonic resonances make them promising candidates for exciton–polariton solar cells, which could extend absorption by combining quantum‐confined 2D states with 3D bandgaps. Both TMD superlattices and 2D perovskites have demonstrated (or are projected to achieve) high power densities of ∼44 W/g. Accordingly, future efforts should prioritize high–power‐density tandem structures as a grand challenge. From this perspective, we can address our first query on the best‐suited geometry for solar cells as mentioned in the introduction section. Although TMDs have high exciton binding energy and low PCE compared to 2D perovskites, the TMD/Si tandem solar cell can outperform single junction TMDs or 2D perovskites in terms of specific power density. Thanks to the strong extinction coefficient of the TMDs, which can provide ultrathin absorption (>100 W/g), while Si can handle bulk current generation.

**Excitonic and Polaritonic Lasers**: In excitonic lasers, halide perovskites have primarily shown lasing under pulsed excitation. Sn‐based perovskites exhibit similar thresholds in both 2D and 3D forms; however, more detailed studies are needed to unlock their full potential. By contrast, all TMDs have demonstrated CW‐pumped excitonic lasing, positioning them as strong candidates for electrically driven lasers. TMD heterostructures hosting interlayer excitons or Moiré lattices also enable room‐temperature lasing in the infrared. For efficient exciton–polariton lasing, high‐Q microcavities are essential. In addition to high‐Q cavities, electrically‐driven exciton–polariton lasers require an asymmetric electrode design from a transparent conducting top electrode, preferably indium tin oxide, and a metal as a back electrode for charge injection. Such a configuration will minimize the increase in laser rise time. Under high current densities, the thermal energy generated from the metal/semiconductor interface can degrade the active layer. Introducing thermal sinks or a heat‐dissipating layer at the metal/semiconductor interface or covering the device will protect the active layer from degradation at high current densities and extend the operating time of the lasers. Additionally, the lasing threshold can be reduced by making all the layers smooth to reduce the optical scattering and photon leakage. CW optically pumped polariton lasing has been reported; electrically driven polariton lasers remain limited to III–V semiconductors. Achieving such devices in TMDs and 2D perovskites represents a major opportunity. Reducing linewidth narrowing beyond what is observed in exciton lasers also remains an open challenge. Overall, TMDs and 2D perovskites are promising platforms for next‐generation exciton‐polariton lasing, combining strong light–matter coupling with potential for scalable integration.

**Photodetectors**: Compared to purely excitonic photodetectors, plasmon–polariton and exciton–polariton detectors offer enhanced responsivity, with 2D materials acting as both sensing and cavity layers when integrated with plasmonic structures. Exploring these architectures may enable single‐photon detection in the telecom regime and open avenues for advanced bioimaging applications.

### 5.1 High Power Density and Space‐Compatible Photovoltaics

Low power conversion efficiency in TMDs‐based solar cells can be due to defects in the TMDs and a lack of materials for establishing ohmic electrical contact to extract the charges. Growing high‐quality monolayer TMDs can address the former challenge, which is already focused on by researchers across the globe. Several post‐treatment strategies, such as TFSI acid treatment to fill the chalcogen vacancies, were established. It can also address another major challenge of Fermi level pinning. Selective electrodes for efficient charge extraction can further improve the PCE. On the other hand, the PCE for the perovskites is higher than that of TMDs; however, focusing on stability and reduced ion migration can increase the efficiency and open up new pathways for different optoelectronic applications.

Further, the high specific power densities for TMD‐based materials are promising for developing next‐generation space‐compatible photovoltaics. In particular, TMD/Si with a high specific power density (>100 W/g) emerged as a suitable candidate for this type of application where weight is a major constraint. The tandem configuration by exploitation of absorption from an ultrathin layer of TMDs combined with the Si can balance between performance and mass of the device. Compared to high PCE in 2D perovskites, in TMD/Si‐based tandem, the PCE can be compensated with a higher power‐to‐mass (W/g) ratio, and can be highly promising. Nevertheless, device performance of the TMD/Si‐based tandem cells upon exposure to gamma or high‐energy electromagnetic radiation should be studied to understand the long‐term performance degradation in such unique conditions.

#### 5.1.1 Key Challenges

- Defect states at the interface of different layers in TMDs are one of the major challenges. These defect states act as non‐radiative recombination centers that trap photogenerated carriers, drastically shortening carrier lifetimes and reducing collection efficiency.
- In the case of 2D perovskites, chemical instability is a major challenge. 2D perovskites suffer from inherent chemical instability stemming from moisture‐induced halide ion migration, oxygen‐catalyzed oxidation of lead species, and organic ligand decomposition under light and thermal stress, which results in rapid performance loss.

#### 5.1.2 Most Promising Strategy

- Defect passivation in TMDs using organic polymers or acid treatment can be explored.
- Encapsulation of 2D perovskites using 2D materials (hBN or graphene) to prevent their degradation can be a second layer of protection, in addition to the organic layers in the 2D perovskites.

### 5.2 Electrically Driven Polariton and Spin‐Polariton Devices

Electrically pumped polaritonic devices are focusing toward on‐chip and field‐tunable photonics architecture. For excitonadhm70890polariton‐based lasers, the preliminary step is to change the pumping mode from optical to electrical. Toward this, polaritons in TMDs have shown CW lasing at cryogenic temperatures, as exciton dissociation due to defects, joule heating, and broad linewidth (>1 nm) limits the operation at room temperature. These detrimental effects of exciton dissociation and linewidth broadening can be mitigated by encapsulation strategies and application of a high Q‐cavity, respectively, as demonstrated with hBN encapsulated WS_2_ placed in DBR. The suppression of deleterious effects reduced the threshold, and lasing can be observed at very low pump power. In contrast to TMD, perovskites have demonstrated room temperature polaritonic lasing mostly under pulsed excitation; however, it is of paramount importance to overcome ion migration and mixed phase formation, which reduces the stability and spectral purity. Further, the goal will be develop a transparent electrode and conductive cavity to reduce absorption losses and efficient electrical injection, respectively, and help to realize compact and low threshold lasers. This also makes lasers the most beneficial device for strong coupling.

Moving toward spin‐polaritonic devices, magnetic field control gives one extra degree of freedom to tune the optoelectronic properties, which will be advantageous for applications such as reconfigurable non‐reciprocity, signal routing, and memory and logic. Combining magnon to polaritonic vdW systems or vdW magnets with an on‐chip photonic resonator can reduce the damping and increase cooperativity to realize compact transducers for spintronics circuits and quantum applications.

#### 5.2.1 Key Challenges

- The synthesis of high‐quality, defect‐free 2D active materials remains a fundamental bottleneck for realizing polariton and spin‐polariton devices, as native point defects (chalcogen vacancies, anti‐site defects) and grain boundaries introduce deep trap states that broaden exciton linewidths, reduce oscillator strength, and dramatically suppress Rabi splitting visibility.
- Designing high‐Q optical cavities compatible with 2D materials presents formidable engineering challenges, as achieving quality factors exceeding 10^2^ (required for strong coupling at room temperature) demands nanometer‐scale precision in layer thickness, atomically smooth interfaces, and minimal absorption losses.
- Absorption loss due to electrical contacts and defects degrades the Q‐factor of the cavity while increasing electrical noise, leakage current, and heating. These effects suppress the visibility of strong coupling signatures such as polaritons and Rabi splitting at room temperature.

#### 5.2.2 Most Promising Strategy

- Growing a clean and defect‐reduced surface using advanced techniques such as MOCVD and MBE.
- Using high reflective DBR materials or the use of BIC modes.
- Avoid lossy contacts and use of transparent current injection layers, for instance, graphene.

### 5.3 Correlative Characterization and Inverse Design

The correlative characterization can unravel the influence of strain, twist angles in the Moiré lattice, and surface roughness toward strong coupling in 2D materials. These parameters significantly impact the Rabi splitting under the strong coupling regime. Current methods limit this correlation between materials features and the polaritonic signatures. The goal can be to develop experimental methods that can measure these parameters non‐destructively with very high accuracy. Further, based on inverse design, we can optimize the elemental framework, such as cavity design and composition, to achieve a specific outcome in the polaritonic system. Using the Gross–Pitaevskii equation can help in accomplishing this step. By combining both the characterization and the calculated model can verify the feasibility of a device for realizing the target applications.

#### 5.3.1 Key Challenges

- Sub‐nanometer correlative characteMoirization of 2D materials faces fundamental probe incompatibilities: optical microscopy limited to ∼50–200 nm (insufficient for 0.3 nm atomic features); electron microscopy causes severe beam damage; scanning probes (AFM/STM, ∼0.1 nm resolution) require ultrahigh vacuum and preclude real‐time picosecond‐timescale polariton dynamics tracking. Correlation between structure and device performance remains impossible without destructive sample preparation.
- Dark exciton formation (with binding energies 10–50 meV below bright excitons in TMDs) cannot be reliably modeled or suppressed, yet their presence alters the effective oscillator strength and dephasing rates of bright excitons through exchange interactions and phonon‐mediated scattering. This makes experimentally measured cavity Q‐factors and Rabi splitting unpredictable from first‐principles calculations. Similarly, surface roughness (substrate disorder and interfacial states) introduces disorder‐induced broadening and a change in oscillator strength, which are extremely difficult to model quantitatively due to the requirement of full atomistic simulation of millions of atoms combined with coupled cluster or GW corrections, computationally challenging for cavity design optimization.

#### 5.3.2 Most Promising Strategy

- A multimodal measurement setup by integrating more than one highly sensitive setup.
- Using different models in conjunction to capture the true nature of the phenomenon and the device.

Although in this review article we focused on 2D‐based systems, researchers have demonstrated optoelectronic devices using relatively thicker layers of the same 2D materials. Despite the indirect bandgap nature in multilayer WS_2_, the device can achieve CW lasing at room temperature under continuous‐wave excitation via phonon‐assisted indirect transitions [349]. Bulk photovoltaics is another major area that is exploiting the bulk form of 2D materials. These results reflect the possibility of device fabrication without delaying the complexity of 2D material synthesis and fabrication.

## Funding

S.B.A. acknowledges the funding support for this work from the New Faculty Initiation Grant (RF23240771MMNFIG009054) by the Indian Institute of Technology Madras and the Prime Minister Early Career Research Grant (ANRF/ECRG/2024/002241/ENS) funded by Anusandhan National Research Foundation. K.S. acknowledges the Institute Postdoctoral Fellowship supported by the Indian Institute of Technology Madras and Visvesvaraya Post Doctoral Fellowship (SP25261446MMMEIT009054) funded by the Ministry of Electronics and Information Technology (MeitY), Government of India. K.D. acknowledges the Summer Research Fellowship supported by the Indian Institute of Technology Madras.

PK acknowledges the funding supported by the National Research Foundation, Singapore, under its NRFF program Award NRF‐NRFF17‐2025‐0113 and by the Nanyang Assistant Professorship program.

[Correction added on 14 February 2026 after first online publication: Funding statement for National Research Foundation is added.]

## Conflicts of Interest

The authors declare no conflicts of interest.

## Data Availability

The authors have nothing to report.
